# The CDK1/TFCP2L1/ID2 cascade offers a novel combination therapy strategy in a preclinical model of bladder cancer

**DOI:** 10.1038/s12276-022-00786-0

**Published:** 2022-06-21

**Authors:** Jinbeom Heo, Jinyoung Lee, Yun Ji Nam, YongHwan Kim, HongDuck Yun, Seungun Lee, Hyein Ju, Chae-Min Ryu, Seon Min Jeong, Jinwon Lee, Jisun Lim, Yong Mee Cho, Eui Man Jeong, Bumsik Hong, Jaekyoung Son, Dong-Myung Shin

**Affiliations:** 1 Department of Biomedical Sciences, Asan Medical Center, University of Ulsan College of Medicine, Seoul, Korea; 2 Department of Physiology, Asan Medical Center, University of Ulsan College of Medicine, Seoul, Korea; 3 Department of Urology, Asan Medical Center, University of Ulsan College of Medicine, Seoul, Korea; 4 Center for Cell Therapy, Asan Medical Center, Seoul, Korea; 5 Department of Pathology, Asan Medical Center, University of Ulsan College of Medicine, Seoul, Korea; 6 Department of Pharmacy, College of Pharmacy, Jeju National University, Jeju, Korea; 7 Interdisciplinary Graduate Program in Advanced Convergence Technology and Science, Bio-Health Materials Core-Facility Center and Practical Translational Research Center, Jeju National University, Jeju, Korea

**Keywords:** Oncogenesis, Cancer stem cells, Bladder cancer, Cancer stem cells

## Abstract

Aberrant activation of embryogenesis-related molecular programs in urothelial bladder cancer (BC) is associated with stemness features related to oncogenic dedifferentiation and tumor metastasis. Recently, we reported that overexpression of transcription factor CP2-like protein-1 (TFCP2L1) and its phosphorylation at Thr177 by cyclin-dependent kinase-1 (CDK1) play key roles in regulating bladder carcinogenesis. However, the clinical relevance and therapeutic potential of this novel CDK1-TFCP2L1 molecular network remain elusive. Here, we demonstrated that inhibitor of DNA binding-2 (ID2) functions as a crucial mediator by acting as a direct repressive target of TFCP2L1 to modulate the stemness features and survival of BC cells. Low *ID2* and high *CDK1* expression were significantly associated with unfavorable clinical characteristics. TFCP2L1 downregulated *ID2* by directly binding to its promoter region. Consistent with these findings, ectopic expression of *ID2* or treatment with apigenin, a chemical activator of ID2, triggered apoptosis and impaired the proliferation, suppressed the stemness features, and reduced the invasive capacity of BC cells. Combination treatment with the specific CDK1 inhibitor RO-3306 and apigenin significantly suppressed tumor growth in an orthotopic BC xenograft animal model. This study demonstrates the biological role and clinical utility of *ID2* as a direct target of the CDK1-TFCP2L1 pathway for modulating the stemness features of BC cells.

## Bladder cancer: Slowing tumor growth by suppressing the suppressor

Combination therapy with apigenin, a powerful antioxidant found in plants such as parsley and camomile, and a drug that inhibits the cell cycle protein CDK1 shows promise for developing therapies for bladder cancer (BC). Switching on genes usually activated in stem cells can cause cancer, including BC. Although CDK1 was known to activate one of these genes in BC cells, no way to suppress the activation had been identified. Jinbeom Heo at University of Ulsan College of Medicine, South Korea, and coworkers investigated CDK1’s role in BC. They found that the transcription factor activated by CDK1 suppressed a protein, ID2, that suppressed stem cell-like characteristics. Simultaneously suppressing CDK1 and boosting ID2 with apigenin strongly repressed tumor growth in a mouse model. These results help point the way to developing new treatment options for BC patients.

## Introduction

Bladder cancer (BC), the tenth most common cancer worldwide, is characterized by high incidence and mortality rates, with 573,278 new cases and >212,536 deaths in 2020^1,2^. BC has a high level of clinical and pathological heterogeneity characterized by high rates of somatic mutations^1^. The high tumor neoantigen burden associated with these alterations led to FDA approval of immunotherapy targeting the checkpoint molecules programmed cell death protein-1/programmed cell death ligand-1 for the treatment of advanced BC^3^. Despite recent advances in the diagnosis and treatment of BC, currently available treatments such as surgery, chemotherapeutic drugs (methotrexate, vincristine, doxorubicin, cisplatin, and cytosine), and biological therapies (Bacillus Calmette–Guérin immunotherapy and immunologic and inactivated bacterial solutions) have provided limited benefits, due mainly to the high recurrence and mortality rates of BC as well as the high costs, adverse effects, and various complications of treatment^4,5^. The poor understanding of the underlying pathogenetic mechanism impedes the development of novel treatment approaches to overcome the limitations of current therapies for BC.

Transcription factors (TFs) and their specific interactions with target genes are crucial for the activation of gene expression programs that determine cell fate and behavior from early embryonic development to tissue homeostasis in adulthood^6^. A subset of TFs that are specifically expressed in pluripotent stem cells, including OCT4 and SOX2, is aberrantly activated in several tumors, including BC^7,8^. In a recent machine learning study, 12,000 samples of 33 tumor types from The Cancer Genome Atlas (TCGA) were analyzed. The results indicated that stemness features are characterized by enrichment of pluripotency-associated TFs and are related to oncogenic dedifferentiation and tumor metastasis^9^. This finding suggests that improving our understanding of the critical determinants of stemness features in BC cells and the clinical relevance of these stemness determinants is critical for identifying novel prognostic markers and developing effective BC therapies.

We recently reported that transcription factor CP2-like protein-1 (TFCP2L1), a pluripotency-associated TF, is phosphorylated at Thr177 by cyclin-dependent kinase-1 (CDK1) in embryonic stem cells and BC cells. TFCP2L1 Thr177 phosphorylation is a central mechanism involved in regulating cell cycle progression and stemness features, which are important for bladder carcinogenesis^10^. Multiplex immunostaining of bladder tumor specimens from 400 patients revealed that a high level of TFCP2L1 and CDK1 coexpression is associated with unfavorable clinical characteristics, including high tumor grade, lymphovascular invasion, muscularis propria invasion, and distant metastasis, and is also an independent prognostic factor for cancer-specific survival. However, the clinical relevance of this novel CDK1-TFCP2L1 molecular network and its therapeutic potential remain to be determined.

In our previous report, silencing of *TFCP2L1* or ectopic expression of a Thr177 phosphorylation-null mutant of *TFCP2L1* induced the expression of differentiation genes, including genes in the bone morphogenetic protein (BMP), GATA, and inhibitor of DNA binding (ID) families, which stimulate urothelial differentiation^10^. Pharmacological activation of the BMP pathway with low-dose FK506 inhibited tumor progression in a murine BC model established by chemical carcinogenesis^11^. In this study, we demonstrated that ID2, a member of the cell differentiation-related helix-loop-helix (HLH) ID family, is a direct repressive target of TFCP2L1 and is involved in modulating the stemness features and survival of BC cells. In human bladder tumors, the expression of ID2 and CDK1 is inversely related, and the combination of low ID2 and high CDK1 expression is associated with unfavorable clinical characteristics, including high tumor grade and muscularis propria invasion. RO-3306, a specific inhibitor of CDK1, enhanced the effects of apigenin, an ID2 activator, in an orthotopic xenograft animal model. The findings of this study suggest that combination treatment with a CDK1 inhibitor and apigenin is a potential therapeutic option for the subgroup of BC patients with high CDK1 and low ID2 expression.

## Materials and methods

### Analysis of clinical cohorts of BC patients

Two independent clinical cohorts of BC patients from TCGA (https://cancergenome.nih.gov) were used in this study. The first cohort was obtained from a comprehensive, integrated study of 131 high-grade muscle-invasive bladder carcinomas (MIBCs)^12^, and the second cohort was obtained from a multiplatform analysis of 412 MIBC patients^13^. Survival data, clinicopathological features, including tumor stage and grade, and gene expression data were obtained from the UCSC Xena Browser (http://xena.ucsc.edu/). Tumor/nontumor differential expression analysis and pairwise gene expression correlation analysis results were analyzed using Gene Expression Profiling Interactive Analysis (GEPIA; http://gepia.cancerpku.cn/index.html), which is an interactive web-based tool that delivers fast and customizable functionalities based on TCGA and Genotype-Tissue Expression (GTEx; https://www.gtexportal.org/) datasets^14^. Kaplan–Meier survival analysis based on high (red) and low (blue) expression levels of ID family genes was performed using Prism 7.0 in the second TCGA study of BC patients^13^. Gene expression datasets from this TCGA cohort were used for differential or pairwise gene expression analysis according to tumor stage and grade.

### Cell culture

The human BC cell lines T24, 5637, HT1197, HT1376, and RT4 were maintained in Eagle’s minimum essential medium (HT1197 and HT1376), McCoy’s 5a (modified) medium (T24 and RT4), and RPMI-1640 medium (5637) (ATCC, Manassas, VA, USA) supplemented with 10% heat-inactivated FBS (HyClone, Pittsburgh, PA, USA) and penicillin/streptomycin (Cellgro, Pittsburgh, PA, USA). Cells were treated with apigenin, 4-hydroxychalcone, and diosmetin (Sigma–Aldrich, Burlington, MA, USA) for 24 h to activate *ID2* expression. To inhibit the activity of CDK1, cells were treated with RO-3306 or CGP74514A (Sigma–Aldrich).

### Ectopic expression and gene silencing

For ectopic expression or silencing of genes of interest, the corresponding open reading frames (ORFs) or specific shRNA constructs were inserted into the pLEX307 (Addgene, plasmid #41392) and pLenti6/Block-iT lentiviral vectors (Invitrogen, Waltham, MA, USA), respectively. pDONR223_ID2_WT_V5 for the human *ID2* ORF was a gift from Jesse Boehm, Matthew Meyerson & David Root (Addgene plasmid # 82960)^15^. Lentivirus was produced using a four-plasmid transfection system (Invitrogen) and concentrated using an Lenti-X Concentrator Kit (Clontech, Mountain View, CA, USA) as previously described^16–18^. Gene expression and functional assays were performed 4 days after lentiviral infection. The information for each ORF and the target sequences for each shRNA are listed in Supplementary Tables 1 and 2, respectively.

### Chromatin immunoprecipitation (ChIP) assay and gene expression analysis

The human *TFCP2L1* ORF was inserted into the pCMV_3Tag-1 vector (Agilent Technologies, Santa Clara, CA, USA)^10^, which was transfected into HT1197 and HT1376 BC cells using Lipofectamine 2000 (Invitrogen). Twenty-four hours after transfection, cross-linked chromatin isolated from cell extracts (1 × 10^7^ cells) was sheared with a Bioruptor Plus sonicator (Diagenode Inc., Denville, NJ, USA) using standard settings (four 20 s pulses at 30 s rest intervals on ice). The ChIP assay was performed using a Magna ChIP G Kit (Millipore, Billerica, MA, USA) as previously described^10,19^.

Real-time quantitative PCR (RT-qPCR) was performed to quantify gene expression. Total RNA was extracted using an RNeasy RNA Isolation Kit (QIAGEN, Valencia, CA, USA), and 50 ng of total RNA was reverse transcribed using Taqman Reverse Transcription Reagents (Applied Biosystems, Foster City, CA, USA). The cycle threshold (*C*t) values were used to determine the relative expression levels of the target genes using the 2^-ΔΔCt^ method as previously described^20,21^. The expression of *GAPDH* was used as the endogenous control. The primers used in the ChIP assay and gene expression analysis are listed in Supplementary Tables 3 and 4, respectively.

### Western blot analysis

Cell extracts (30 μg) were prepared in RIPA lysis buffer (Santa Cruz Biotechnology, Santa Cruz, CA, USA) supplemented with protease and phosphatase inhibitor cocktail (Roche, Indianapolis, IN, USA) and separated on 12% SDS–PAGE gels. The expression levels of the indicated proteins were assessed by incubation with the following antibodies: anti-ID2 (NBP-88630; NovusBio), anti-TFCP2L1 (OAAB09732; Aviva Systems Biology, San Diego, CA, USA), anti-CDK1 (sc-54; Santa Cruz Biotechnology), anti-PARP (9542; Cell Signaling), anti-cleaved caspase-3 (9661; Cell Signaling), anti-β-actin (A5441; Sigma–Aldrich), and anti-Flag epitope (F3165; Sigma–Aldrich).

### Immunocytochemical analysis

For immunocytochemistry, human BC cells fixed with 4% paraformaldehyde (Sigma–Aldrich) were stained with antibodies against ID2 (M1301-2, HUABIO, Woburn, MA, USA) or TFCP2L1 (OAAB09732; Aviva Systems Biology) prior to visualization using Alexa Fluor 488-conjugated (A11001) anti-mouse and Alexa Fluor 546-conjugated (A11010) anti-rabbit antibodies (Molecular Probes, Grand Island, NY, USA), respectively. The stained samples were imaged using an inverted fluorescence microscope (EVOS® FL Color Imaging System, Life Technologies, Carlsbad, CA, USA).

### Cell proliferation and apoptosis assays

The cell proliferation capacity was determined by an MTT assay (Sigma–Aldrich). Apoptotic cell death was analyzed using an Annexin-V fluorescein isothiocyanate (FITC)/propidium iodide (PI) assay. Cells were harvested by trypsinization, washed with PBS, resuspended in Annexin-V binding buffer (10 mM HEPES (pH 7.4), 140 mM NaCl, and 2.5 mM CaCl_2_), and labeled with Annexin-V FITC and PI. The FITC- and/or PI-labeled cell populations were quantified and analyzed on a flow cytometer (Beckman-Coulter, Brea, CA, USA).

### Tumor sphere formation and limiting dilution assays

For the tumor-sphere formation assay, BC cells were resuspended as a single cell suspension in a 1:1 ratio of serum-free keratinocyte growth medium (Gibco, Waltham, MA, USA) and growth factor-reduced Matrigel (BD Biosciences, Mountain View, CA, USA) and then plated into ultra-low-attachment plates (Costar, Corning, NY, USA). The size of the tumor spheres was measured 7 days after the first plating. For quantification, the perimeter of the tumor spheres was measured in eight randomly chosen representative areas selected from each group using ImageJ software (National Institute of Mental Health, Bethesda, MD, USA).

For the limiting dilution assay, BC cells were diluted to a density of 1 cell per well and plated into 96-well plates in 50 μl of culture medium. Fresh culture medium was added every 2 days, and the plated cells were cultured for 10 days after plating. The number of colonies was calculated for quantitative analysis.

### In vitro cell invasion assay

In the upper chambers of Transwell permeable supports (Corning Inc.), the 8.0 μm pore polycarbonate membrane filters were precoated with Matrigel (BD Biosciences) diluted 1:5 in serum-free DMEM. BC cells were seeded in the upper chambers at 2 × 10^4^ cells/well in 100 μl of serum-free DMEM, and culture medium supplemented with 3% FBS was added to the lower chambers. Cell invasion ability was assessed by counting the number of cells that had migrated to the lower side of the membrane after incubation at 37 °C in a 5% CO_2_ incubator for 24 h. Three randomly chosen visual fields (magnification, 200×) in each Transwell chamber were used for quantitative analysis.

### Orthotopic implantation of BC cells (xenograft model)

All animal experiments in this study were approved by the Institutional Animal Care and Use Committee of the University of Ulsan College of Medicine (IACUC-2020-12-209). Eight-week-old male NOD/ShiLtJ-*Prkdc*^*em1AMC*^*Il2rg*^*em1AMC*^ (NSGA) mice were purchased from GEM Biosciences Inc. (Cheongju, Republic of Korea). After 1 week of adaptation in the Asan Medical Center laboratory animal facility, the mice were implanted with 1.0 × 10^6^ HT1376 BC cells in a volume of 100 μl by direct injection into the outer layer of the anterior wall and dome of the bladder using a 500 μm syringe and a 26-gauge needle, as previously reported^10,22^. Three weeks after orthotopic implantation of BC cells, mice were intraperitoneally injected with RO-3306 (4 mg/kg) and apigenin (50 mg/kg) alone or in combination via six injection cycles at 4-day intervals. The mice and injection sites were monitored every 2 days for 45 days after the initial implantation of BC cells. At the endpoint, tumors were obtained by dissection for measurement of tumor size and histological examination or immunofluorescence staining. Mice were randomly allocated to treatment groups (*n* = 5 or *n* = 10 per group), and the order of cell transplantation, treatment, and evaluation as well as daily examinations were randomized. Investigators involved in the tumor size measurements and histological assessments were blinded to the treatment groups.

### Histological examination

For histological analysis, the bladders of xenograft mice were fixed with 4% paraformaldehyde for 24 h. After cryoprotection in 30% sucrose for 24 h, each bladder was sliced into 20 μm sections using a cryostat (Leica, Lussloch, Germany) and stained with hematoxylin and eosin (H&E). For immunofluorescence (IF) staining, bladders were stained with antibodies against ID2 (NBP-88630; NovusBio), TFCP2L1 (OAAB09732; Aviva Systems Biology), CDK1 (ab131450; Abcam, Cambridge, MA, USA), CD44 (ab78960; Abcam), and cytokeratin 14 (KRT14; ab7800; Abcam). Alexa Fluor 488-conjugated (A11001 and A11008) anti-mouse and anti-rabbit antibodies or an Alexa Fluor 546-conjugated anti-rabbit antibody (A11010) were used as secondary antibodies (Molecular Probes). Nuclei were counterstained with 4′,6-diamino-2-phenylindole (DAPI; D9542; Sigma–Aldrich). Three representative areas per slide were randomly selected for each animal. The stained samples were imaged using an inverted fluorescence microscope (EVOS® FL Color Imaging System, Life Technologies).

### Statistics

Quantitative results were statistically analyzed using the nonparametric Mann–Whitney *U*-test or one- or two-way ANOVA with the Bonferroni post hoc test. All analyses were performed using GraphPad Prism 7.0 software (GraphPad Software, La Jolla, CA, USA), with *p* < 0.05 considered statistically significant.

## Results

### ID2 is a direct target of TFCP2L1 in BC cells

**Fig. 1:**
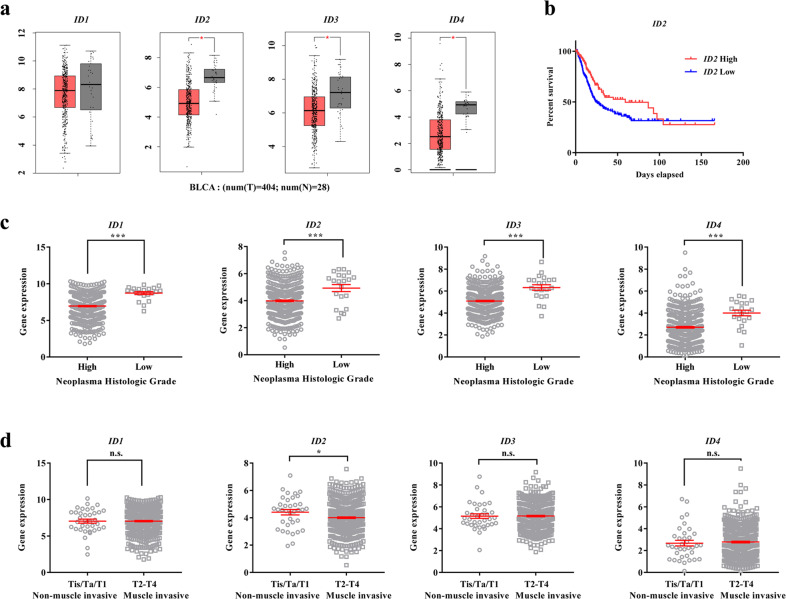
Repression of ID family genes in the TCGA study of BC patients. **a** Dot plots showing the expression of ID family genes (*ID1*−*4*) in normal (N; *n* = 28) and bladder tumor (T; *n* = 404) samples from a TCGA dataset^13^ of BC patients analyzed using GEPIA (http://gepia.cancer-pku.cn/). **b** Kaplan–Meier analysis of the survival of BC patients stratified by high (red) and low (blue) expression levels of *ID2* in the TCGA dataset. **c**, **d** Expression of ID family genes in subgroups of BC patients from the TCGA study according to tumor grade (**c**) and pT (**d**) tumor stage. Raw gene expression data were obtained from the UCSC Xena Browser (http://xena.ucsc.edu/). Quantitative results are presented as the mean ± SEM values. Statistical analyses were performed using the nonparametric Mann–Whitney *U*-test. *p < 0.05, ***p < 0.001, n.s. not significant. The exact P values and numbers of biological replicates can be found in the source data.

In both embryonic stem and BC cells, TFCP2L1 repressed the expression of differentiation genes, including those in the BMP, GATA, and ID families^10^. Considering the favorable effect of BMP activation on inhibiting tumor progression^11^, we investigated the clinical relevance of these urothelial differentiation genes in BC. Analysis of TCGA datasets of BC patients^12,13^ using the GEPIA^14^ and UCSC Xena (http://xena.ucsc.edu/) web servers revealed that a subset of TFCP2L1 target genes, including *BMP2*, *BMP4*, *BMP5*, *GATA6*, and *SOX17*, was significantly downregulated in bladder tumors relative to normal urothelium (Supplementary Fig. 1a). However, the expression of most of these genes barely changed with BC tumor grade, although GATA6 was upregulated in patients with high-grade BC tumors (Supplementary Fig. 1b). Regarding the ID family genes, the expression of *ID2*, *ID3*, and *ID4* but not *ID1* was significantly lower in bladder tumors than in normal urothelium (Fig. 1a). Although the expression of ID genes was not significantly related to the overall survival of BC patients in the TCGA datasets (Fig. 1b and Supplementary Fig. 2a, b), their expression was significantly lower in high-grade BC than in low-grade BC (Fig. 1c). In particular, the *ID2* transcript was significantly downregulated in BC patients with higher pT tumor stages indicating bladder muscle invasion (Fig. 1d). We therefore focused on the role of ID2 among the CDK1-TFCP2L1 pathway targets related to urothelial differentiation in BC.

**Fig. 2:**
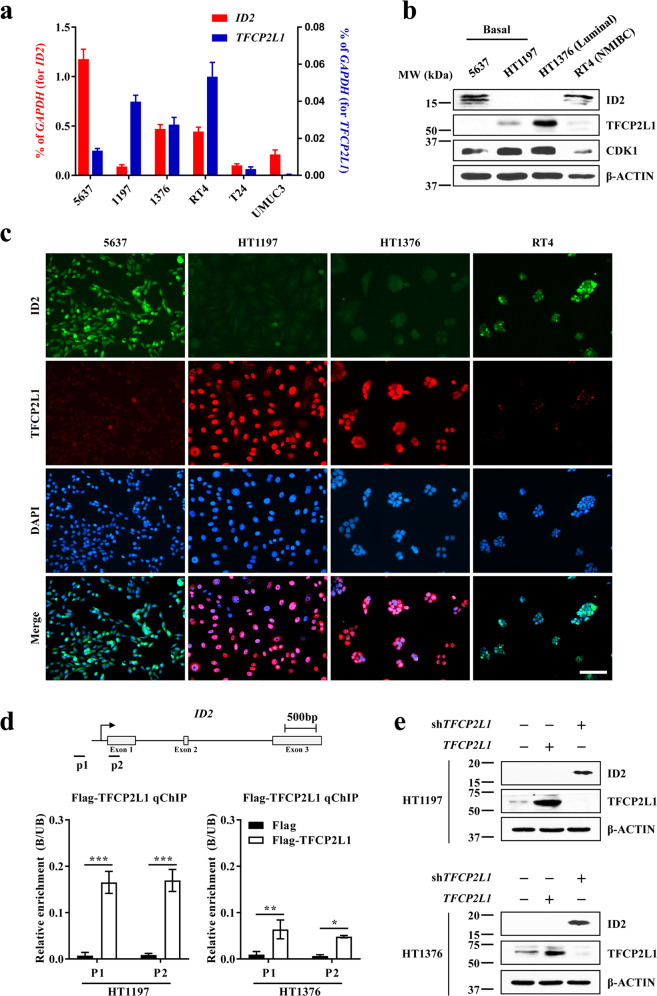
Reciprocal expression of ID2 and TFCP2L1 in human BC cells. **a**–**c** ID2 and TFCP2L1 transcript (**a**) and protein (**b**, **c**) levels in basal (5637 and HT1197), luminal (HT1376), and mixed (T24 and UMUC3) subtypes of muscle-invasive BC (MIBC) as well as in RT4 non-muscle-invasive BC cells. **b** Western blot analysis of ID2, TFCP2L1, and CDK1 in the indicated human BC cell lines. β-Actin was used as the loading control. Molecular weight (MW) marker sizes (in kD) are shown on the left. **c** Representative images of double immunofluorescence staining of ID2 (green) and TFCP2L1 (red) proteins at 200× magnification are shown. Scale bars = 100 µm. Nuclei were stained with DAPI (blue). **d** ChIP–qPCR assay of Flag-TFCP2L1 binding to the promoter region of *ID2* in basal HT1197 and luminal HT1376 muscle-invasive BC (MIBC) cells (*n* = 4). A schematic diagram of the *ID2* locus and the sites recognized by primers used in the ChIP–qPCR assay are shown in the top panel. Quantitative data are expressed as the mean ± SEM values. Statistical analyses were performed using two-way ANOVA with the Bonferroni *post hoc* test. *p < 0.05, ***p < 0.001 compared with the Flag control group. **e** Protein expression of ID2 and TFCP2L1 in the indicated BC cells 4 days after infection with lentiviruses expressing the *TFCP2L1* ORF or shRNA specific for *TFCP2L1* (sh*TFCP2L1*). The exact *P*-values and numbers of biological replicates can be found in the source data.

We next compared the endogenous expression levels of ID2 and TFCP2L1 in human BC cells derived from muscle-invasive BC (MIBC) tumors with different molecular classifications, including basal-like subtype (5637 and HT1197), luminal-like subtype (HT1376)^13,23^, and mixed subtype (T24 and UMUC3) cells, as well as in the RT4 cell line, often used as a model of non-muscle-invasive BC^24^. In most BC cell lines tested, the *ID2* and *TFCP2L1* transcript levels were inversely related (Fig. 2a), and their reciprocal expression was marked at the protein level, as determined by western blotting and immunofluorescence staining (Fig. 2b, c). In particular, the ID2 protein was almost undetectable in the HT1197 and HT1376 BC cell lines, which showed the highest levels of TFCP2L1.

To determine whether TFCP2L1 directly represses the transcription of *ID2*, we performed ChIP assays in HT1197 and HT1376 BC cells expressing Flag-tagged TFCP2L1 (Flag-TFCP2L1) (Supplementary Fig. 3a). ChIP–qPCR analysis showed that TFCP2L1 was bound to the promoter of *ID2* in both BC cell lines (Fig. 2d). Consistent with this result, *TFCP2L1* knockdown restored the expression of *ID2* (Fig. 2e and Supplementary Fig. 3b, c), whereas overexpression of TFCP2L1 downregulated *ID2* (Fig. 2e). These results indicate that ID2 is a direct repressive target of TFCP2L1 in human BC cells. We previously reported that TFCP2L1 overexpression stimulated the tumorigenic potency of human BC cells in a xenograft model^10^. In line with the results shown in Fig. 2e, ID2 protein expression was barely detectable in xenograft tumors with *TFCP2L1* overexpression; however, it was increased in xenograft samples with *TFCP2L1* silencing (Supplementary Fig. 3d), further supporting the hypothesis that TFCP2L1 negatively regulates ID2 expression.

### ID2, a direct target of TFCP2L1, suppresses the stemness features of BC cells

**Fig. 3:**
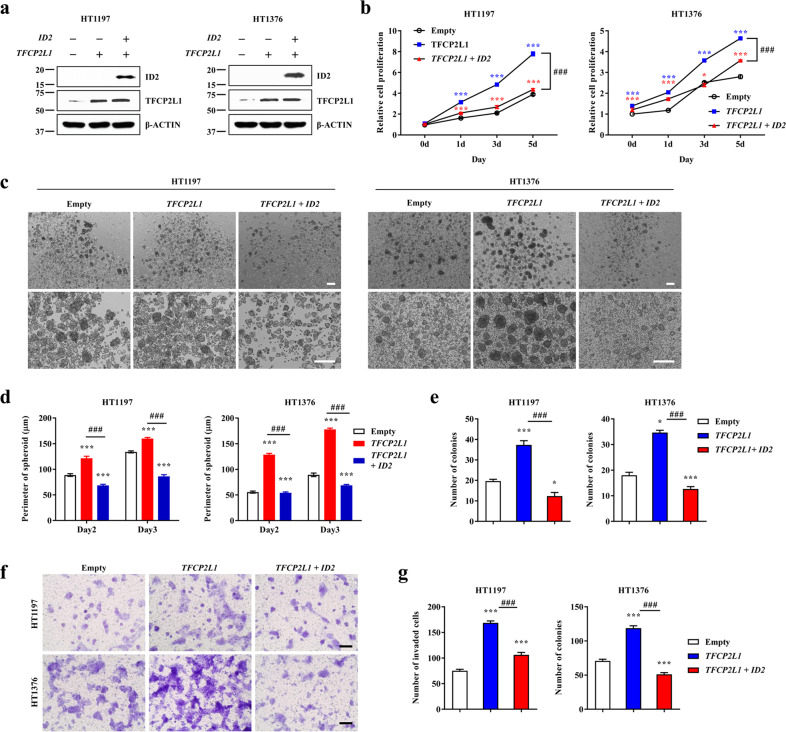
ID2 suppressed the TFCP2L1-mediated stemness features of BC cells. **a** Ectopic expression of the TFCP2L1 or ID2 protein was validated by western blot analysis in basal (HT1197) and luminal (HT1376) MIBC cell lines. **b**–**g** Cell proliferation (**b**, *n* = 4), tumor-sphere formation (**c**, **d**; *n* = 10), clonogenic limiting dilution (**e**, *n* = 3), and Matrigel invasion (**f**, **g**, *n* = 6) capacities of HT1197 and HT1376 BC cells 4 days after overexpression of *TFCP2L1* alone or in combination with *ID2* (*TFCP2L1* + *ID2*). Representative images of the tumor-sphere formation and Matrigel invasion assays are shown at 40× (upper panel in **c**), 100× (lower panel in **c**), or 200× (**f**) magnification. Scale bars = 100 µm. Lentivirus expressing empty vector was used as the control. All quantitative data are expressed as the mean ± SEM values. Statistical analyses were performed using one-way (**e–g**) or two-way (**b–d**) ANOVA with the Bonferroni *post hoc* test. *p < 0.05, ***p < 0.001 compared with the empty control group; ^##^*p* < 0.001 compared with the TFCP2L1 group. The exact P values and numbers of biological replicates can be found in the source data.

Next, we investigated whether ID2 affects the proliferation or stemness features of BC cells, both of which are positively modulated by the CDK1-TFCP2L1 pathway^10^. Ectopic expression of *ID2* alone decreased the proliferation of HT1197 and HT1376 BC cells, which have low levels of endogenous *ID2* expression (Supplementary Fig. 4a). Consistent with these findings, silencing of *ID2* increased the proliferation of 5637 and RT4 cells, which have high levels of endogenous *ID2* expression (Supplementary Fig. 4b, c), supporting a crucial role of ID2 in the division of BC cells. In particular, forced expression of *ID2* significantly prevented the increase in proliferation induced by *TFCP2L1* overexpression in HT1376 and HT1197 BC cells (Fig. 3a, b).

The stemness features of BC cells were examined by assessing tumor-sphere formation and the clonogenic capacity. Overexpression of *ID2* suppressed the increase in tumor-sphere formation induced by *TFCP2L1* overexpression (Fig. 3c, d). A clonogenic limiting dilution assay showed that *ID2* inhibited TFCP2L1-induced self-renewal activity (Fig. 3e). The results of the Transwell invasion assay indicated that HT1376 and HT1197 BC cells overexpressing *TFCP2L1* had a significantly higher invasive potential than control cells and that *ID2* overexpression suppressed the increase in their invasive capacity (Fig. 3f, g). In line with these findings, silencing of *ID2* significantly promoted the stemness features of 5637 and RT4 cells, based on their tumor-sphere formation, clonogenic, and invasion capacities (Supplementary Fig. 4d–g). Taken together, these results indicate that ID2 is a negative downstream target of TFCP2L1 involved in modulating the growth, stemness features, and invasiveness of BC cells.

### Efficacy of apigenin treatment for suppressing BC tumorigenesis

**Fig. 4:**
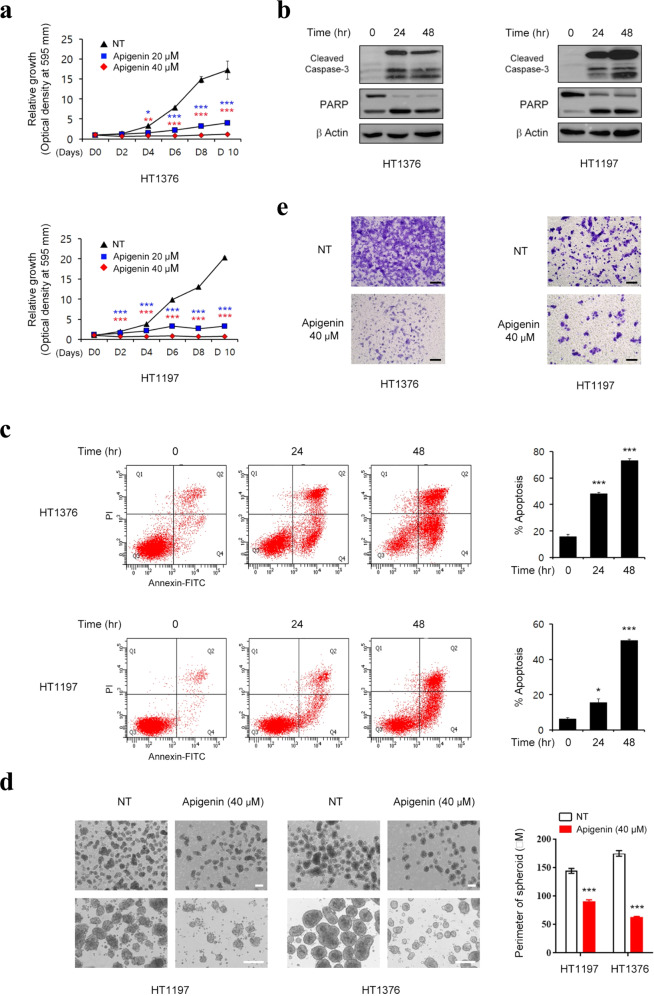
In vitro efficacy of apigenin treatment for suppressing BC tumorigenesis. **a** HT1376 (*n* = 6) and HT1197 (*n* = 4) BC cells were treated with the indicated concentrations of apigenin, and cell growth was assessed. **b** BC cells were treated with apigenin (40 µM) for up to 48 h and immunoblotted with the indicated antibodies. **c** BC cells were treated with apigenin (40 µM) for up to 48 h, and cell death was assessed by Annexin-V/PI staining and flow cytometry. The quantitative assessments of apoptotic cells (Annexin-V^+^/PI^−^ population, %; *n* = 3) are shown in the panels to the right of the representative flow cytometry plots. **d**, **e** Tumor-sphere formation (**d**, *n* = 10) and Matrigel invasion (**e**) assays of the indicated human BC cells after treatment with apigenin (40 µM) for 24 h. Representative images are shown at 40× (upper panel in **d**), 100× (lower panel in **d**), or 200× (**e**) magnification. Scale bars = 100 µm. NT indicates the nontreated control group. Quantitative data are expressed as the mean ± SEM values. Statistical analyses were performed using one-way (**c**) or two-way (**a**–**d**) ANOVA with the Bonferroni post hoc test. *p < 0.05, ***p < 0.001 compared with the empty control group. The exact P values and numbers of biological replicates can be found in the source data.

To determine whether ID2 activation can be therapeutically targeted in BC, we used apigenin, a nontoxic dietary flavonoid that activates BMP signaling and enhances the expression of ID2^25^. Treatment with apigenin significantly decreased BC cell growth in a dose-dependent manner (Fig. 4a) and activated the effector caspase-3 and cleavage of poly-(ADP-ribose) polymerase (PARP) in BC cells (Fig. 4b). Consistent with these findings, apoptotic cell death was activated in a time-dependent manner upon treatment with apigenin (Fig. 4c). Apigenin markedly suppressed tumor-sphere formation and the invasion of basal and luminal BC cells (Fig. 4d, e). Apigenin could mediate these anticancer activities through multiple signaling pathways, including the PI3K/AKT, MAPK, JAK/STAT, NF-κB and WNT/β-catenin pathways, not just through the ID2 pathway^26^. To isolate the exact mechanism, BMP-activating small molecules such as diosmetin and 4-hydroxychalcone, which upregulate ID2 protein expression, were also tested^25^. Similar to apigenin, both BMP−ID2 activators were able to suppress the growth, tumor-sphere formation, and invasion capacities of HT1376 and HT1197 BC cells, in parallel with the activation of apoptotic cell death (Supplementary Figs. 5 and 6). These data suggest that ID2 activation is a potential new therapeutic approach for BC.

To examine the in vivo relevance of these findings, intraperitoneal injection of apigenin (50 mg/kg, six doses at 4-day intervals) was performed in an orthotopic xenograft model of BC in which HT1376 cells were transplanted into the outer layer of the bladder in NSG immunodeficient mice^10^. The results showed that tumor growth was suppressed by 25.6 ± 6.17% in the apigenin monotherapy group (Supplementary Fig. 7a, b). Histological examination showed that apigenin had little effect on tumor grade (Supplementary Fig. 7c). These results indicate that treatment with apigenin alone has limited in vivo antitumor activity in BC.

### Clinical significance of the reciprocal expression of CDK1 and ID2 in BCs

**Fig. 5:**
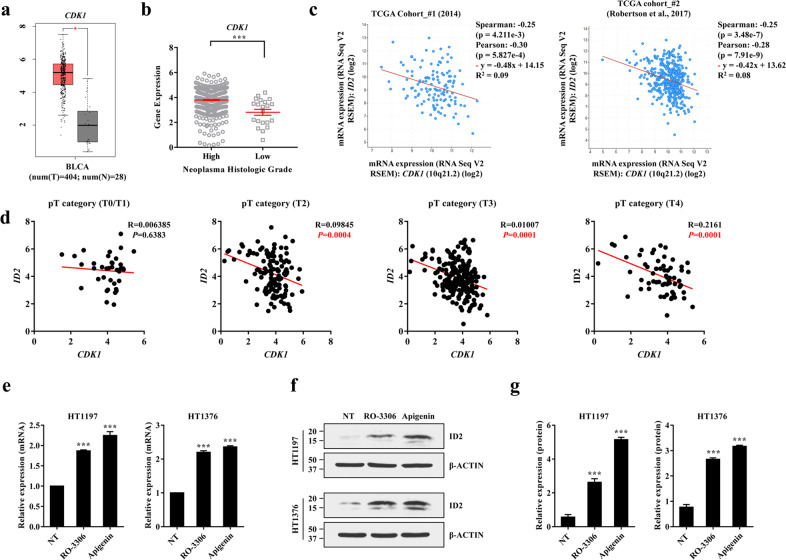
Reciprocal expression of CDK1 and ID2 in human BC cells. **a**–**c** Dot plots showing the expression of *CDK1* in normal (N; *n* = 28) and bladder tumor (T; *n* = 404) samples (**a**), based on tumor grade (**b**), and pairwise gene expression correlations with *CDK1* transcript expression (**c**) after analysis of TCGA datasets of BC patients using the GEPIA web server. Quantitative results are presented as the mean ± SEM values. **b** Statistical analyses were performed using the nonparametric Mann–Whitney *U*-test. ***p < 0.001. **d** Pairwise correlations between *ID2* and *CDK1* transcript levels in pT tumor stage subgroups in a TCGA dataset (Robertson et al., 2017) of BC patients. All gene expression data were obtained from the UCSC Xena Browser (http://xena.ucsc.edu/). **e**–**g** RT-qPCR (**e**) and western blot (**f**, **g**) analyses of the expression of ID2 in basal HT1197 or luminal HT1376 MIBC cells after exposure to 20 μM RO-3306 for 2 h or 40 μM apigenin for 24 h. β-Actin was used as the loading control. Molecular weight (MW) marker sizes (in kD) are shown on the left. **g** Quantification of ID2 protein expression. **e**, **g** Quantitative data are expressed as the mean ± SEM values (*n* = 4). Statistical analyses were performed using one-way ANOVA with the Bonferroni post hoc test. ***p < 0.001 compared with the nontreated (NT) control group.

Considering the limited in vivo efficacy of apigenin therapy, we investigated whether CDK1, an upstream activator of TFCP2L1, can enhance the therapeutic effect of apigenin. First, we evaluated the expression of *CDK1* and its relationship to *ID2* in TCGA cohorts. The expression of *CDK1* was higher in bladder tumors than in normal urothelial tissues (Fig. 5a). Consistent with the expression of *ID2*, the *CDK1* transcript level was dependent on tumor grade in BC, and *CDK1* was upregulated in high-grade bladder tumors (Fig. 5b). In line with these results, a high expression level of *CDK1* correlated with decreased *ID2* expression in two independent TCGA cohorts^12,13^ (Fig. 5c). Other ID family genes also showed a reciprocal expression pattern with respect to CDK1 (Supplementary Fig. 8a, b). The inverse relationship between *CDK1* and *ID2* expression was significantly associated with pT stage (Fig. 5d and Supplementary Fig. 9a–c). Importantly, treatment with RO-3306, similar to treatment with apigenin, increased the expression of *ID2* in HT1376 and HT1197 BC cells at both the transcript (Fig. 5e) and protein (Fig. 5f, g) levels. Induction of ID2 expression was also observed after treatment with 4-hydroxychalcone or CGP74514A^27^, another CDK1-specific inhibitor (Supplementary Fig. 10). Collectively, these results suggest a potential association between *CDK1* and *ID2* and the clinical significance of this association in BC patients.

### Efficacy of apigenin and RO-3306 for BC treatment

**Fig. 6:**
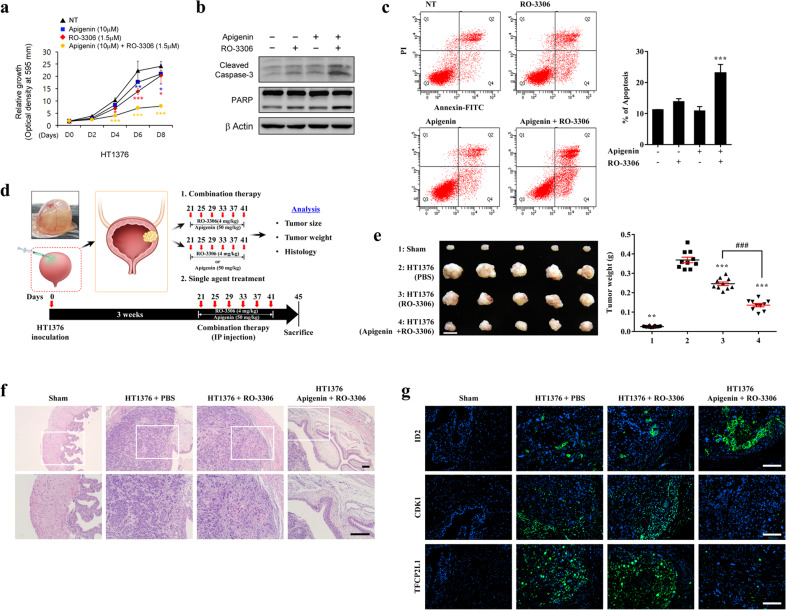
Combination treatment of BC based on the reciprocal expression of CDK1 and ID2. **a** BC cells were treated with the indicated concentrations of apigenin and RO-3306, and cell growth was assessed (*n* = 3). NT indicates the nontreated control group. **b**, **c** BC cells were treated with suboptimal concentrations of apigenin (15 µM) and RO-3306 (3 µM) for up to 48 h. Cell death was assessed by (**b**) immunoblotting of cleaved caspase-3 and PARP and by (**c**) Annexin-V/PI staining and flow cytometry. The quantitative assessments of apoptotic cells (Annexin-V + /PI− population, %; *n* = 3) are shown in the panels to the right of the representative flow cytometry plots. Quantitative data are expressed as the mean ± SEM values. Statistical analyses were performed using one-way (**c**) or two-way (**a**) ANOVA with the Bonferroni post hoc test. *p < 0.05, **p < 0.01, ***p < 0.001 compared with the NT control group. **d** Experimental overview of the orthotopic BC xenograft model generated by transplantation of 1.0 × 10^6^ human HT1376 BC cells through the outer layer of the bladder of nonobese diabetic (NOD) Cg-*Prkdc*^scid^ *Il2rg*^*tm1Wjl*^/SzJ (NSG) immunodeficient mice. Treatment with RO-3306 alone or in combination with apigenin was performed by intraperitoneal injection according to the indicated schedules. **e** Representative images (left panel) and weights (right panel) of bladders bearing tumors after treatment with RO-3306 (4 mg/kg) alone or combined with apigenin (50 mg/kg) in duplicate experiments (five mice in each replicate). The data are shown as dot plots of the mean ± SEM values from ten independent animals in each group. ***p < 0.001 compared with the vehicle control group; ^###^p < 0.001, one-way ANOVA with the Bonferroni *post hoc* test. **f** Representative images of hematoxylin and eosin staining of bladder tissues from the indicated xenograft groups at 100× (upper panel) or 200× (lower panel) magnification. Scale bars = 100 µm. **g** Immunofluorescence assay for detecting ID2, CDK1, and TFCP2L1 (green) proteins in xenograft tumors. Representative merged images are shown at 200× magnification. Scale bars = 100 µm. Nuclei were stained with DAPI (blue).

The association between *CDK1* and *ID2* prompted us to explore the therapeutic effects of a CDK1 inhibitor alone or in combination with apigenin using in vitro cellular and in vivo BC xenograft models. First, we examined whether RO-3306, a CDK1 inhibitor, has a synergistic effect with apigenin in the treatment of BC. When apigenin or RO-3306 was used at a suboptimal concentration, apigenin significantly impaired cell growth only when combined with RO-3306 (Fig. 6a). Consistent with this result, treatment with either apigenin or RO-3306 alone at a suboptimal concentration did not have a significant effect on the cleavage of caspase-3 and PARP, whereas treatment with the combination of apigenin and RO-3306 caused caspase-3 and PARP cleavage and induced apoptosis (Fig. 6b, c).

Next, we tested the effect of RO-3306 alone or in combination with apigenin in the orthotopic BC xenograft mouse model (Fig. 6d). As shown in Fig. 6e, tumor growth was inhibited by 33.2 ± 2.48% and 63.1 ± 2.31% in the RO-3306 monotherapy and RO-3306 plus apigenin groups, respectively. Histological examination of xenograft bladder tumors confirmed the synergistic antitumor effect of RO-3306 and apigenin combination therapy, showing marked reductions in bladder tumor size and progression (Fig. 6f). Vehicle-treated and RO-3306 monotherapy-treated xenograft tumors showed low ID2 protein levels but upregulation of CDK1 and TFCP2L1 (Fig. 6g), along with BC stemness feature markers, including CD44 and KRT14 (Supplementary Fig. 11). In contrast, combination treatment with RO-3306 and apigenin markedly suppressed CDK1-TFCP2L1–mediated stemness features by activating ID2 expression (Fig. 6g and Supplementary Fig. 11). Taken together, these results indicate that CDK1 inhibition enhances the therapeutic efficacy of apigenin, suggesting that this combination can overcome the limited effect of apigenin and improve outcomes in subgroups of BC patients.

## Discussion

In this study, we demonstrated the biological role and clinical utility of *ID2* as a direct target of the CDK1-TFCP2L1 pathway for modulating the stemness features of BC cells. All ID proteins, including ID2, heterodimerize with interacting proteins, including class I (E proteins such as E12, E47, HEB, and E2-2) and class II (e.g., the tissue-specific myogenic regulatory factor-like protein MyoD) basic HLH TFs, retinoblastoma protein (pRB) and related pocket proteins, paired box TFs, and the S5a subunit of the 26 S proteasome^28^. Because they lack a DNA-binding motif, ID proteins function as dominant-negative inhibitors of interacting protein-driven transcription by forming nonfunctional heterodimers, thus playing a critical role in the regulation of cell cycle progression and cell differentiation^28^. Dysregulated expression of ID proteins is associated with tumor growth, vascularization, invasiveness, metastasis, and chemoresistance, as well as stemness features, in a wide range of tumors.

Emerging evidence supports the important role of ID2 in many cancers; however, ID2 expression has distinct effects in different tumor types. In multiple myeloma, colon adenocarcinoma, neuroblastoma, and prostate cancer, overexpression of *ID2* stimulates pro-oncogenic activities by inhibiting the pRB tumor suppressor pathway, resulting in cell cycle progression, activation of β-catenin–mediated transcription, and overexpression of the antiapoptotic gene B-cell leukemia-3^29–32^. However, ID2 also has antitumor effects. For example, low expression of *ID2* and its associated gene signature are correlated with poor prognosis in MLL-rearranged acute myeloid leukemia, where it acts as a negative regulator of leukemia stem cell potential^33^. Induction of *ID2* expression is required to maintain differentiated and noninvasive phenotypes in acute promyelocytic leukemia^34^ and breast cancer cells^35,36^. A TCGA study of BC showed that *ID2* transcript levels are significantly reduced in the basal subtype, which is the most aggressive subtype of invasive urothelial carcinoma^11^. The different roles of ID2 in different tumor types could be attributed to heterogeneity in the presence and expression levels of its interacting partners, the subcellular localization of ID2, and the expression signature of ID2 transcriptional target genes. Further investigation is necessary to elucidate the precise mechanism of action by which ID2 orchestrates CDK1-TFCP2L1-mediated stemness features in cells of different BC subtypes.

The therapeutic potency of ID2 in BC was examined using BMP signaling activators, including apigenin, which upregulate ID2 expression. Apigenin exerts broad antitumor effects in various types of cancers by (i) preventing cell growth and cell cycle progression, (ii) triggering apoptosis, (iii) modulating autophagy, and (iv) inhibiting cell motility, migration, and invasion^26^. These anticancer properties of apigenin are mediated through multiple signaling pathways, including the PI3K/AKT, MAPK/ERK, JAK/STAT, NF-κB and WNT/β-catenin pathways^26^. The importance of ID2 in the effect of apigenin on BC tumorigenesis was validated by the similar anticancer effects of two independent ID2 inducers mediated by BMP signaling-activating small molecules such as 4-hydroxychalcone and diosmetin. However, the present study has a limitation in that the precise pharmaceutical target of the BMP−ID2 cascade has not been well characterized, making it difficult to discern the mechanism of action underlying the anticancer activities of apigenin, particularly regarding combination therapy with the CDK1 inhibitor RO-3306 for the clinical management of BC. In addition, compounds that specifically target this novel CDK1-TFCP2L1-ID2 oncogenic pathway need to be identified.

Given that BC has high levels of clinical and pathological heterogeneity, a combinatorial therapeutic strategy is necessary for effective cancer therapy. The main purposes of the combinatorial strategy are to potentiate the antitumor effects of chemotherapeutic agents and to overcome the limitations caused by toxicity and acquired drug resistance^37^. Apigenin is an effective anticancer agent, but it exhibits only moderate anticancer efficacy when used alone at physiological dosages in humans^38^. In line with these findings, the in vivo efficacy of apigenin monotherapy showed limited antitumor activity despite its very promising results in in vitro cell model assays. Therefore, cotreatment with other therapeutic agents is a reasonable strategy to enhance the anticancer activities of apigenin.

Analysis of several clinical datasets of BC patients indicated that ID2 showed significant reciprocal expression with CDK1, which has been considered a potential therapeutic target for cancer, and a series of CDK inhibitors have been developed^27^. In particular, CDK1 targeting could be a favorable approach for BC treatment, based on the finding that urothelial carcinoma subtypes with a poor prognosis exhibit high expression levels of late cell cycle genes such as *CDK1* and the cyclin B complex or genes related to chromosome segregation and cell division^39,40^. In the present study, RO-3306, a potent and selective inhibitor of CDK1, effectively enhanced the in vivo antitumor effects of apigenin in an orthotopic xenograft animal model of BC. Consistent with this finding, blocking CDK1 with RO-3306 increased the efficacy of sorafenib and cisplatin treatment in preclinical models of hepatocellular carcinoma and epithelial ovarian cancer, respectively^41,42^. Therefore, a combination therapy targeting the CDK1-TFCP2L1-ID2 oncogenic pathway could provide improved therapeutic feasibility and safety for use in BC patients.

In this study, we demonstrated the biological role and clinical utility of the CDK1-TFCP2L1-ID2 oncogenic pathway for modulating the stemness features of BC cells. The clinical and pathological heterogeneity of BC can result in treatment failure, including high rates of recurrence and poor outcomes in patients with advanced disease^4^. Therefore, it is important to explore the subgroups of BC patients that would benefit from a novel therapeutic strategy targeting the CDK1-TFCP2L1-ID2 oncogenic pathway and to identify the specific molecular biomarkers and molecular signatures for early diagnosis and prediction of the response to this targeted therapy.

## Supplementary information

Supplementary information

Supplementary Dataset S1
