# Supplementary material for: The CDK1/TFCP2L1/ID2 cascade offers a novel combination therapy strategy in a preclinical model of bladder cancer
Source: Exp Mol Med. 2022 Jun 21;54(6):801–11. doi: 10.1038/s12276-022-00786-0 (PMC9256744; doi:10.1038/s12276-022-00786-0)
Supplement: Supplementary file 1 — Supplementary information [file 12276_2022_786_MOESM1_ESM.pdf]

Supplementary Information

The CDK1/TFCP2L1/ID2 cascade offers a novel combination therapy strategy in a preclinical model of bladder cancer

Jinbeom Heo[1], Jinyoung Lee[1], Yun Ji Nam[1], YongHwan Kim, HongDuck Yun, Seungun Lee, Hyein Ju, Chae-Min Ryu, Seon Min Jeong, Jinwon Lee, Jisun Lim, Yong Mee Cho, Eui Man Jeong, Bumsik Hong*, Jaekyoung Son*, Dong-Myung Shin*

[1]These authors contributed equally to this work.

*Corresponding authors: d0shin03@amc.seoul.kr, jaekson@amc.seoul.kr and bshong@amc.seoul.kr

This PDF file includes:

Supplementary Figures 1 to 11 and figure legends

Supplemenatry Tables 1 to 4

Supplementary References

Uncropped western blot results

Other Supplementary Information includes the following (separate file):

Source data for quantification analyses

SUPPLEMENTARY FIGURE LEGENDS

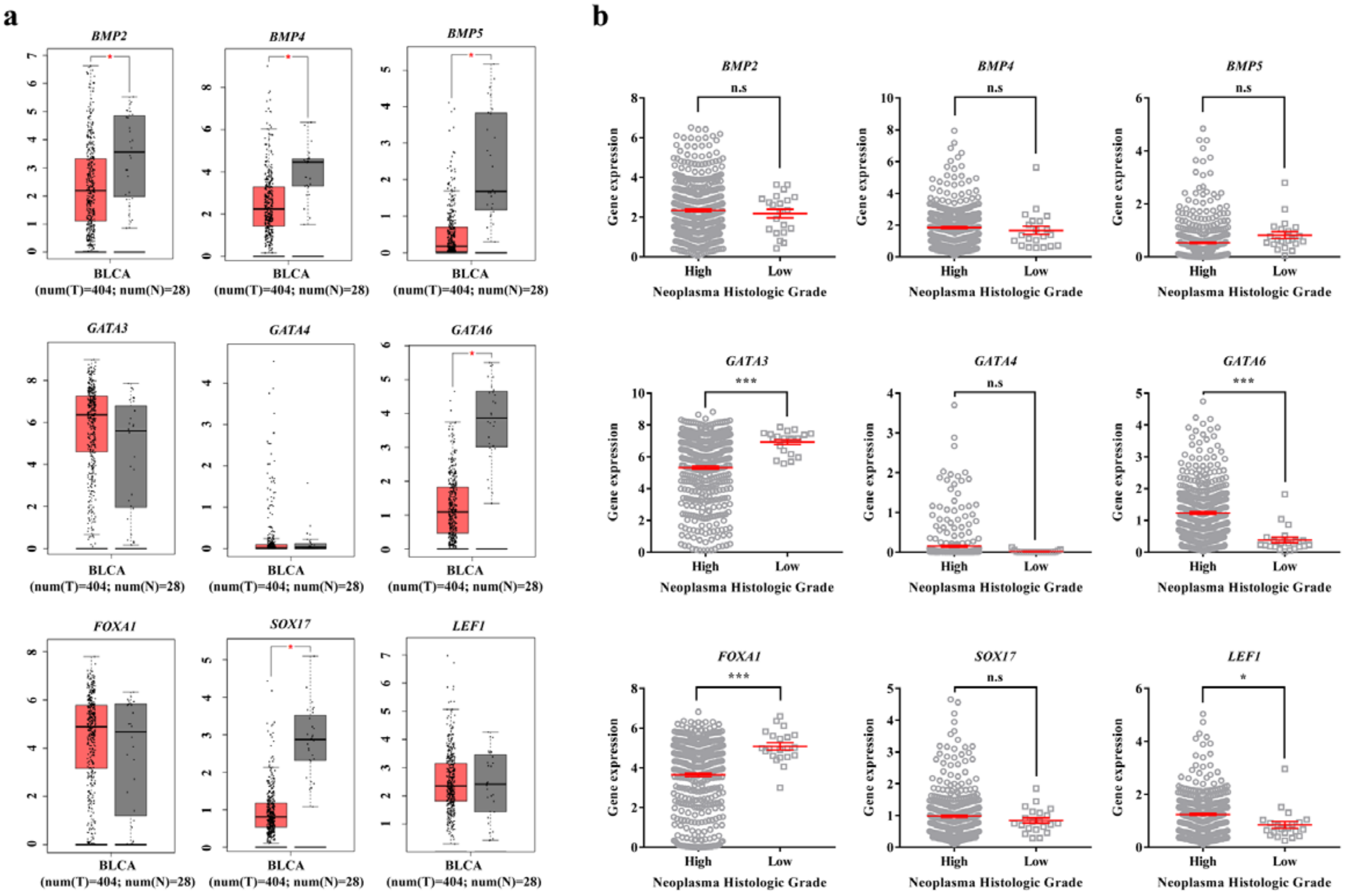

Supplementary Fig. 1 Expression of the urothelial differentiation genes in the TCGA study of BC patients. a Dot plots show the expression of the urothelial differentiation genes including BMP and GATA family genes in normal (N; n = 28) and bladder tumor (T; n = 404) samples from a TCGA dataset [1] of BC patients analyzed using GEPIA (http://gepia.cancer-pku.cn/). Note that these genes were repressed by the CDK1–TFCP2L1 pathway in human BC cells in a previous report [2]. **b** Expression of the urothelial differentiation genes in subgroups of BC patients from the TCGA study according to tumor grade. Gene expression data were obtained from UCSC's Xena project (http://xena.ucsc.edu/). Quantitative results are presented

as the mean ± SEM. Statistical analyses were performed using non-parametric Mann–Whitney U tests. *p < 0.05, ***p < 0.001, n.s. = not significant. The exact P-values and number of biological replicates can be found in the source data.

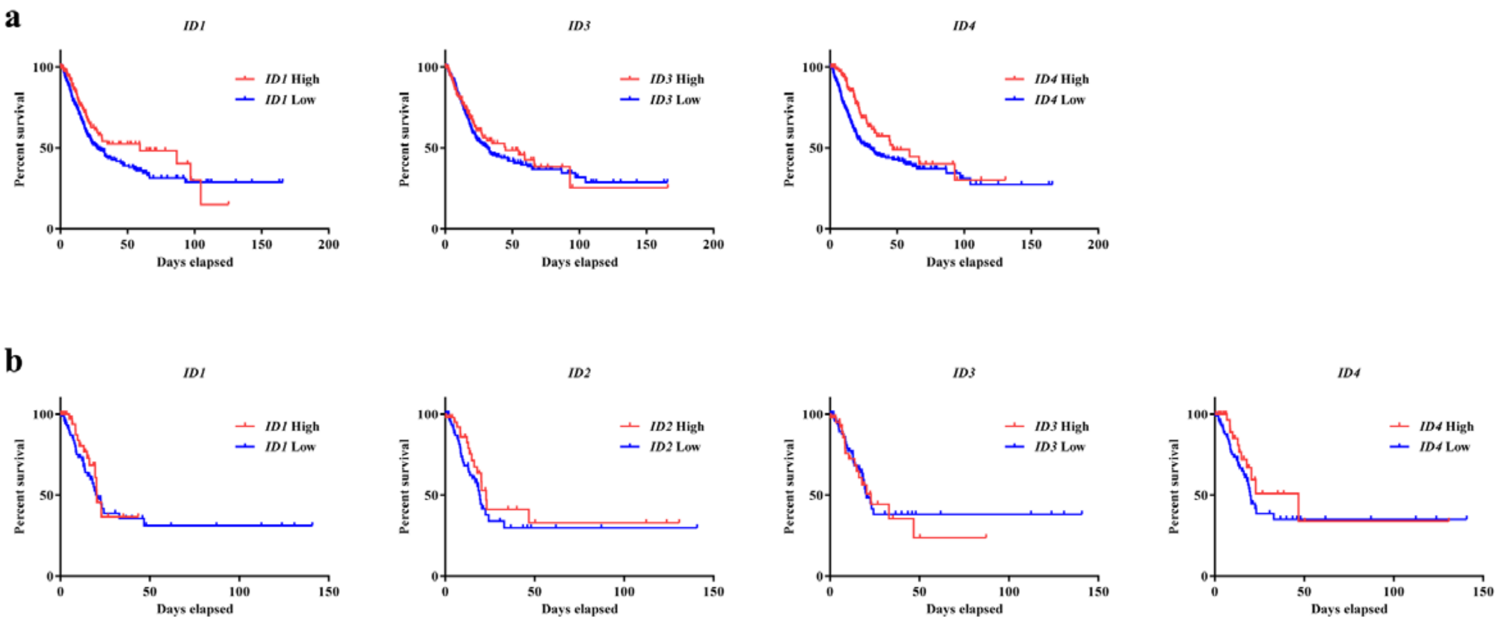

Supplementary Fig. 2 Survival BC patients according to ID family gene expression. a and **b** Kaplan–Meier analysis of the survival of BC patients according to high (red) and low (blue) expression levels of ID family genes (*ID1 – 4*) in the two TCGA datasets. The first TCGA cohort (**a**) was a multiplatform analysis of 412 MIBC patients (Robertson et al., Cell. 2017;171:540-56) and the second one (**b**) included 131 high-grade MIBC (The Cancer Genome Atlas Research Network, Nature. 2014;507:315).

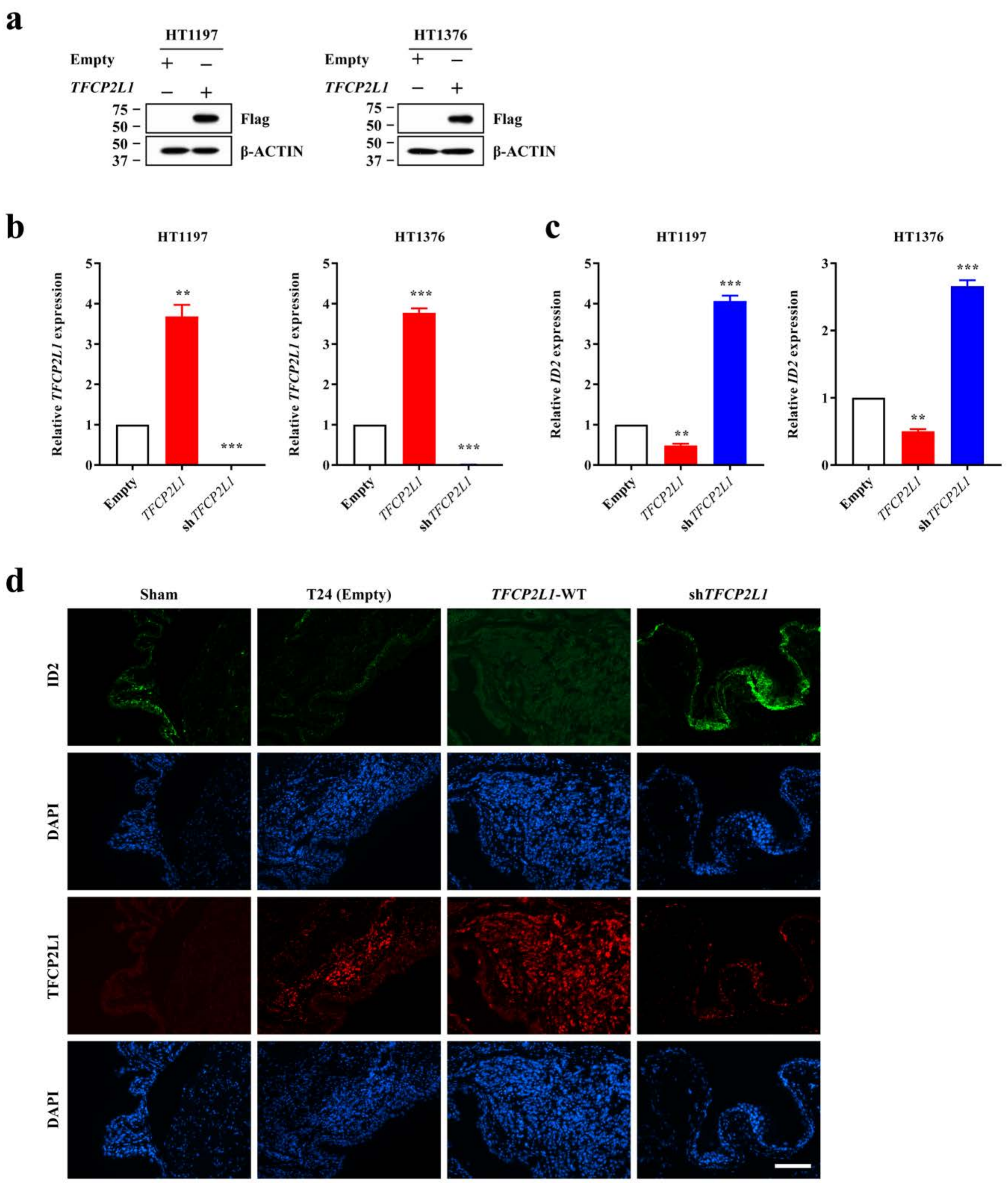

Supplementary Fig. 3 TFCP2L1 suppresses the expression of ID2 in BC cells. a Validation of the expression of Flag-tagged TFCP2L1 (Flag-TFCP2L1) in the indicated BC cells for chromatin immunoprecipitation (ChIP) analysis, which is presented in Fig 2**e**. Empty construct

was used in the control group. **b** and **c** RQ-PCR assays of *TFCP2L1* (**b**) and *ID2* (**c**) expression in the indicated BC cells at 4 days after infection with lentiviruses containing human *TFCP2L1* ORF or shRNA against *TFCP2L1* (sh*TFCP2L1*). All quantitative data are presented as the mean ± SEM (n = 4). Statistical analyses were performed using one-way ANOVA with Bonferroni *post hoc* tests. **p < 0.01, ***p < 0.001 compared with the empty vector control group. **d** Immunofluorescence assay for detecting ID2 (green) and TFCP2L1 (red) proteins in bladders bearing xenograft tumors at 4 weeks after transplantation of T24 human BC cells harboring empty control, *TFCP2L1* ORF, and sh*TFCP2L1* constructs. Representative images are shown at ×200 magnification. Scale bars = 100 µm.

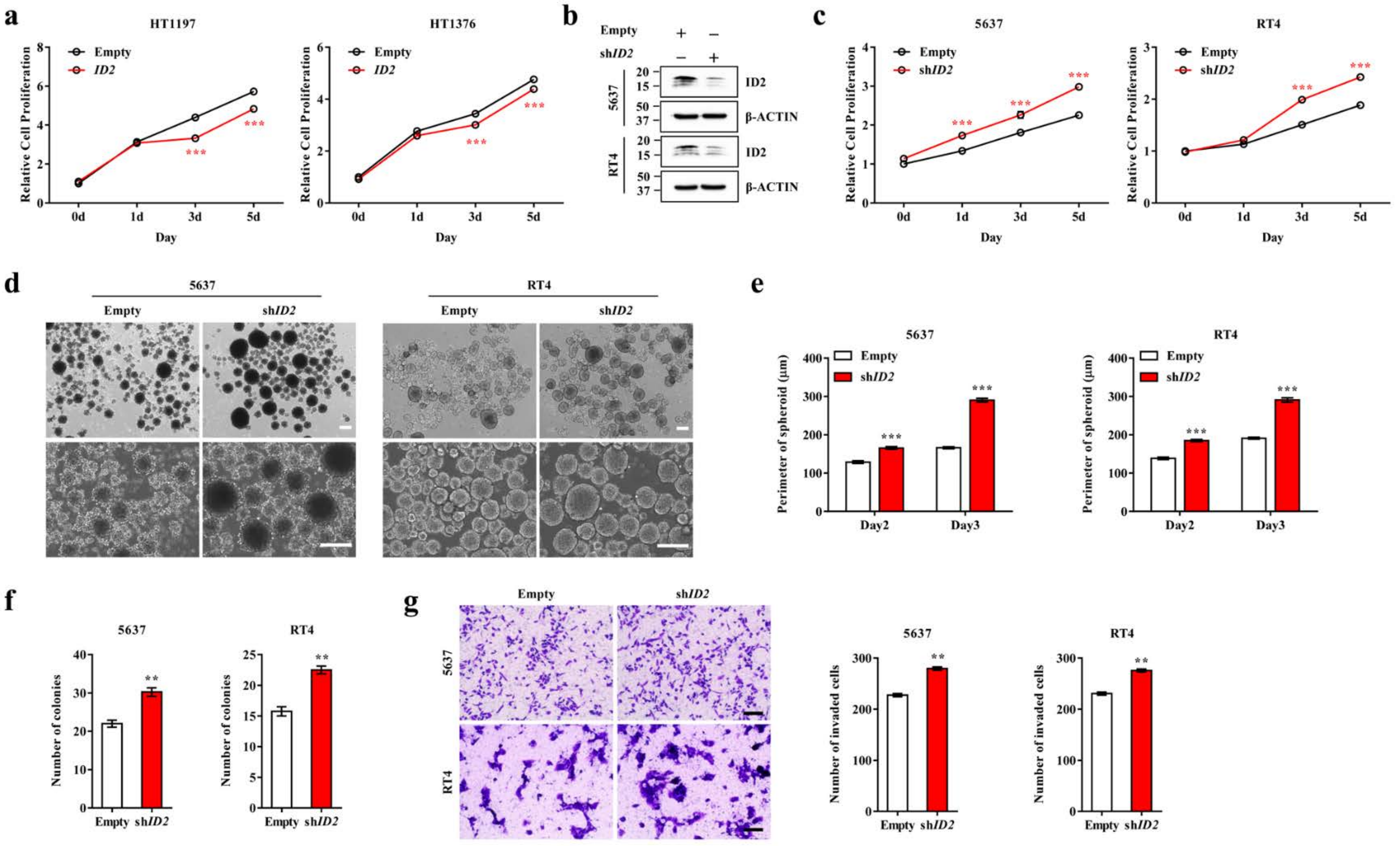

Supplementary Fig. 4 ID2 suppressed the cell growth and stemness features of BC cells. **a** Cell proliferation (n = 4) in basal-like subtype (HT1197) and luminal-like subtype (HT1376) MIBC cell lines at the indicated days after overexpression of *ID2*. **b** Silencing of ID2 protein was validated by western blot analysis in basal-like subtype MIBC (5637) and non-muscle-invasive BC (RT4) cell lines. β-actin was used as a loading control. Molecular weight (MW) marker sizes (kD) are shown on the left. **c-g** Cell proliferation (**c** n = 4) and tumor sphere formation (**d**, **e** n = 10), clonogenic limiting dilution (**f** n = 3), and Matrigel invasion (**g** n = 6) capacities of 5637 and RT4 BC cells at 4 days after expression of the *ID2* specific shRNA (sh*ID2*). Representative images for tumor sphere formation and Matrigel invasion assays are shown at ×40 (upper panel in **d**), ×100 (lower panel in **d**), or ×200 (**g**) magnification. Scale bars

= 100 μm. Lentivirus containing an empty construct was used as the control. All quantitative data are expressed as the mean ± SEM. Statistical analyses were performed using one-way (**e-g**) or two-way (**a** and **c**) ANOVA with Bonferroni *post hoc* tests. *p < 0.05, **p < 0.01, ***p < 0.001 compared with empty control group. The exact P-values and number of biological replicates can be found in the source data.

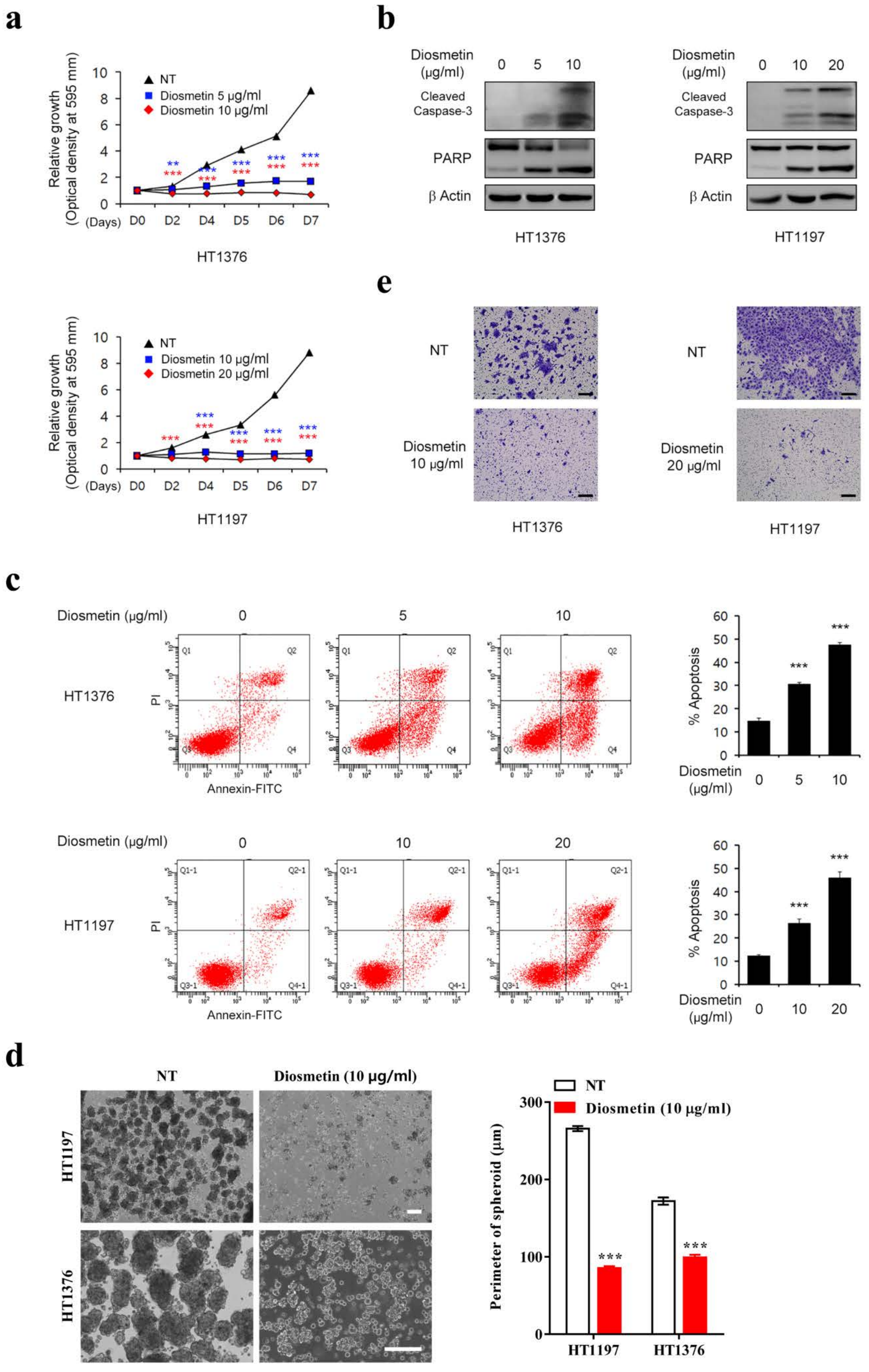
a
NT
Diosmetin 5 μg/ml
Diosmetin 10 μg/ml
Relative growth (Optical density at 595 mm)
(Days) D0 D2 D4 D5 D6 D7
HT1376
NT
Diosmetin 10 μg/ml
Diosmetin 20 μg/ml
HT1197
b
Diosmetin (μg/ml) 0 5 10
Cleaved Caspase-3
PARP
β Actin
HT1376
Diosmetin (μg/ml) 0 10 20
HT1197
e
NT
Diosmetin 10 μg/ml
Diosmetin 20 μg/ml
c
Diosmetin (μg/ml) 0 5 10
HT1376
PI
Annexin-FITC
% Apoptosis
Diosmetin (μg/ml) 0 10 20
HT1197
d
NT
Diosmetin (10 μg/ml)
Perimeter of spheroid (μm)
HT1197
HT1376

Supplementary Fig. 5. Diosmetin exerts inhibitory effects on BC tumorigenesis. a HT1376 (n = 3) and HT1197 (n = 3) BC cells were treated with the indicated concentrations of diosmetin, and cell growth was assessed. **b** BC cells were treated with the indicated concentrations of diosmetin for up to 24 h and immunoblotted with the indicated antibodies. **c** BC cells were treated with the indicated concentrations of diosmetin for up to 24 h, and cell death was assessed by Annexin-V/PI staining and flow cytometry. The results of quantification of apoptotic cells (% of Annexin-V^+/PI^- population, n = 3) are shown in the right panels of the representative flow cytometry diagrams. **d** and **e** Tumor sphere formation **(d** n = 10**)** and Matrigel invasion **(e)** assays of the indicated human BC cells after treatment with the indicated concentrations of Diosmetin for 24 h. Representative images are shown at ×40 (upper panel in **d**), ×100 (lower panel in **d**), or ×200 (**e**) magnification. Scale bars = 100 µm. N.T. indicates not-treated control. Quantitative data are expressed as the mean ± SEM. Statistical analyses were performed using one-way **(c)** or two-way **(a and d)** ANOVA with Bonferroni *post hoc* tests or *p < 0.05, ***p < 0.001 compared with the empty control group. The exact P-values and number of biological replicates can be found in the source data.

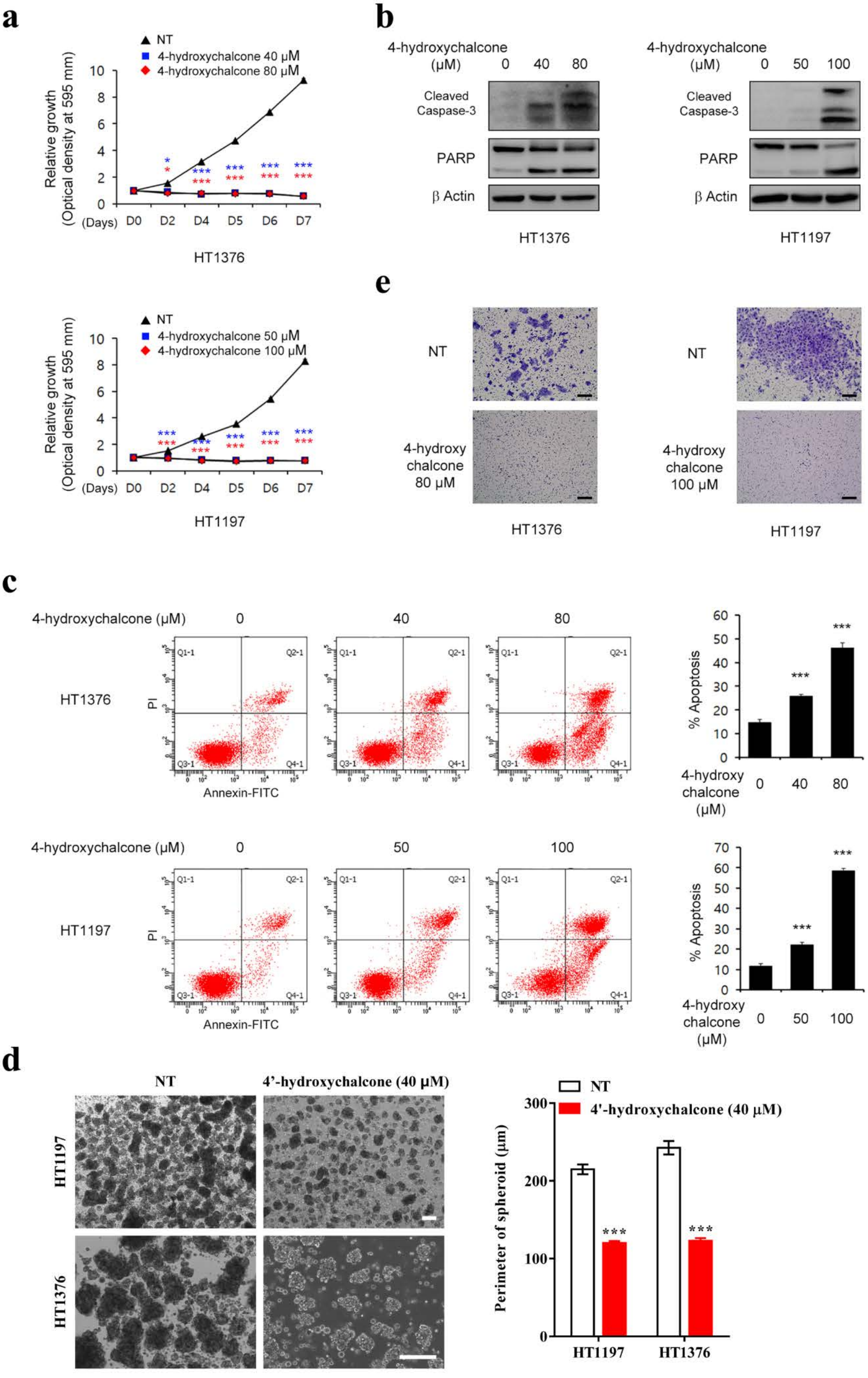
a
NT
4-hydroxychalcone 40 μM
4-hydroxychalcone 80 μM
Relative growth (Optical density at 595 mm)
(Days) D0 D2 D4 D5 D6 D7
HT1376
NT
4-hydroxychalcone 50 μM
4-hydroxychalcone 100 μM
Relative growth (Optical density at 595 mm)
(Days) D0 D2 D4 D5 D6 D7
HT1197
b
4-hydroxychalcone (μM) 0 40 80
Cleaved Caspase-3
PARP
β Actin
HT1376
4-hydroxychalcone (μM) 0 50 100
Cleaved Caspase-3
PARP
β Actin
HT1197
e
NT
4-hydroxy chalcone 80 μM
HT1376
NT
4-hydroxy chalcone 100 μM
HT1197
c
4-hydroxychalcone (μM) 0 40 80
HT1376
PI
Annexin-FITC
% Apoptosis
4-hydroxy chalcone (μM) 0 40 80
4-hydroxychalcone (μM) 0 50 100
HT1197
PI
Annexin-FITC
% Apoptosis
4-hydroxy chalcone (μM) 0 50 100
d
NT
4'-hydroxychalcone (40 μM)
HT1197
HT1376
NT
4'-hydroxychalcone (40 μM)
Perimeter of spheroid (μm)
HT1197 HT1376

Supplementary Fig. 6. Inhibitory effects of 4-hydroxychalcone on BC tumorigenesis. a HT1376 (n = 3) and HT1197 (n = 3) BC cells were treated with the indicated concentrations of 4-hydroxychalcone and cell growth was assessed. **b** BC cells were treated with the indicated concentrations of 4-hydroxychalcone for up to 24 h and immunoblotted with the indicated antibodies. **c** BC cells were treated with the indicated concentrations of 4-hydroxychalcone for up to 24 h, and cell death was assessed by Annexin-V/PI staining and flow cytometry. The results of quantification of apoptotic cells (% of Annexin-V^+/PI^- population, n = 3) are shown in the right panels of the representative flow cytometry diagrams. **d** and **e** Tumor sphere formation **(d** n = 10**)** and Matrigel invasion **(e)** assays of the indicated human BC cells after treatment with the indicated concentrations of 4-hydroxychalcone for 24 h. Representative images are shown at ×40 (upper panel in **d**), ×100 (lower panel in **d**), or ×200 (**e**) magnification. Scale bars = 100 µm. N.T. indicates not-treated control. Quantitative data are expressed as the mean ± SEM. Statistical analyses were performed using one-way **(c)** or two-way **(a and d)** ANOVA with Bonferroni *post hoc* tests or *$p < 0.05$, ***$p < 0.001$ compared with the empty control group.

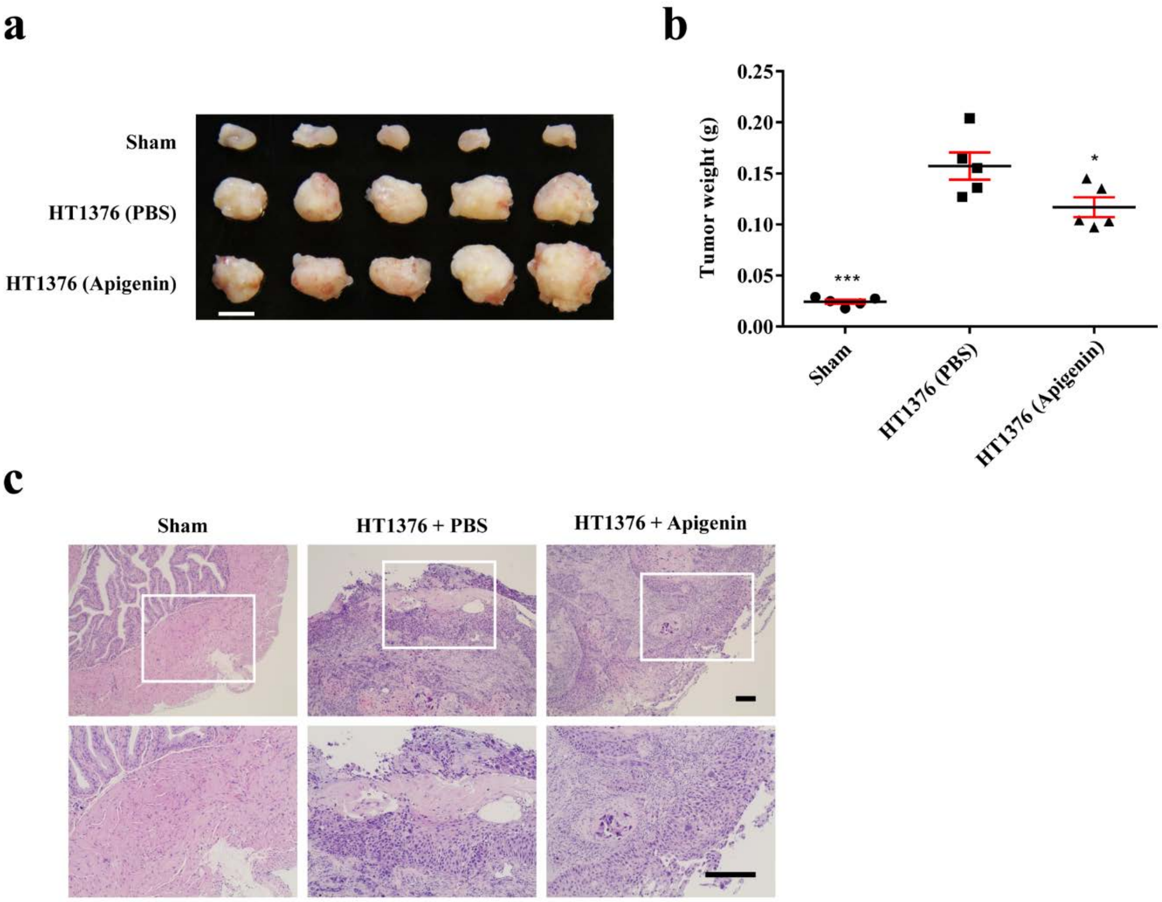

Supplementary Fig. 7 Limited *in vivo* efficacy of apigenin treatment in BC. a and **b** Representative images (**a**) and weight (**b**) of bladders bearing tumors at the endpoint after administration of apigenin (50 mg/kg) single treatment, which was initiated at 3 weeks after BC cell transplantation with six cycles at 4 day intervals (five mice per group). Data are shown as dot plots of the mean ± SEM. *p < 0.05, ***p < 0.001 compared with PBS vehicle control, one-way ANOVA with the Bonferroni *post hoc* test. **c** Hematoxylin and eosin staining of bladder tissues from the indicated xenograft groups. Representative images are shown at ×100 (upper panel) or ×200 (lower panel) magnification. Scale bars = 100 μm.

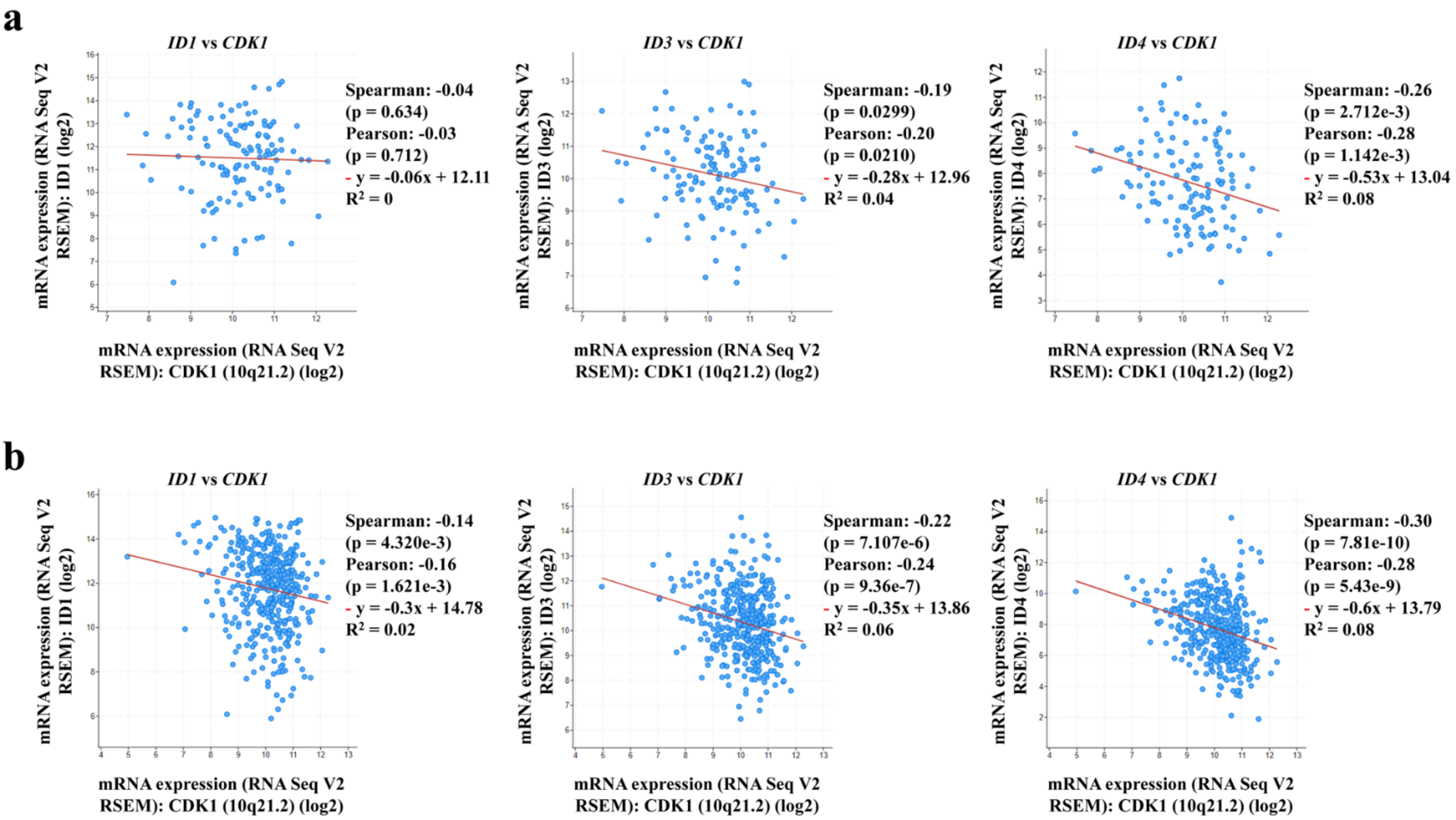

Supplementary Fig. 8 Clinical association between CDK1 and ID family genes in BC. a and **b** Pairwise gene correlation between *CDK1* and ID family genes including *ID1*, *ID2*, and *ID4* in two independent TCGA datasets of BC patients. The first TCGA cohort (**a**) included 131 high-grade MIBC (The Cancer Genome Atlas Research Network, Nature. 2014;507:315) and the second one (**b**) was a multiplatform analysis of 412 MIBC patients (Robertson et al., Cell. 2017;171:540-56). The results were extracted from the cBioPortal webserver (https://www.cbioportal.org/).

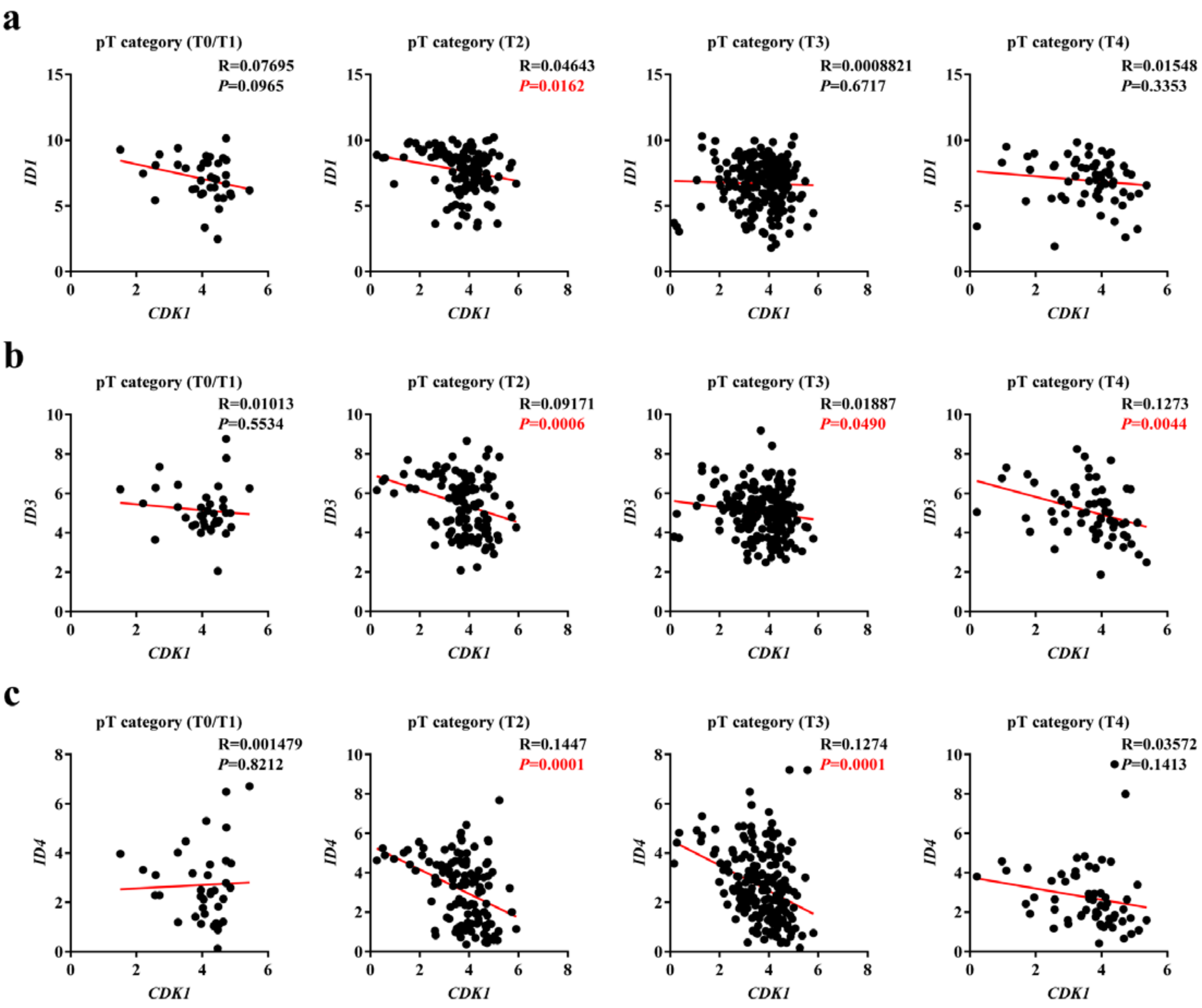

Supplementary Fig. 9 Clinical significance of CDK1 and ID family gene expression in aggressive BCs. a-c Pairwise gene correlation between *CDK1* and ID family genes including *ID1* (**a**), *ID3* (**b**), and *ID4* (**c**) in subgroups of BC patients according to pT stage in the TCGA study (Robertson et al., Cell. 2017;171:540-56). Gene expression data were obtained from UCSC's Xena project (http://xena.ucsc.edu/). Note that the inverse relationship between *CDK1* and ID family genes was significant in subgroups of BC patients with >T2 grade in which the tumor had spread to the bladder muscle.

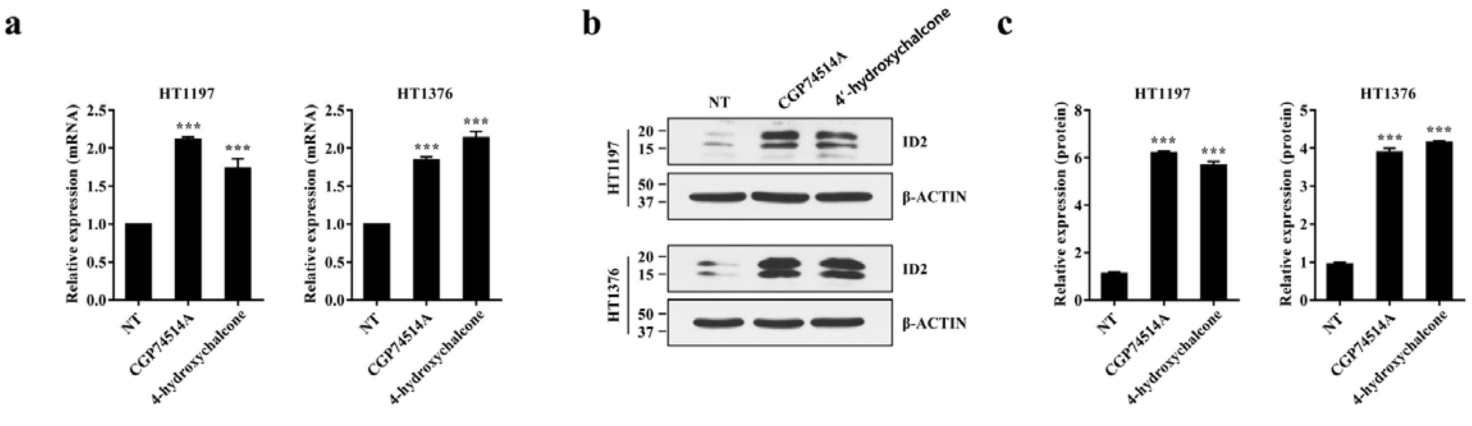

Supplementary Fig. 10 Induction of ID2 by CDK1 inhibitor and ID2 activator. a-c RQ-PCR **(a)** and western blot **(b** and **c)** analysis for the expression of ID2 in basal-like HT1197 or luminal-like HT1376 subtypes MIBC cells after exposure to 5 μM CGP74514A or 15 μM 4-hydroxychalcone for 24 hours. β-actin was used as a loading control. Molecular weight (MW) marker sizes (kD) are shown on the left. **(c)** The quantification data of ID2 protein. Quantitative data are expressed as the mean ± SEM (n = 4). Statistical analyses were performed using one-way ANOVA with Bonferroni *post hoc* tests. ***$p < 0.001$ compared with the non-treated (NT) control group.

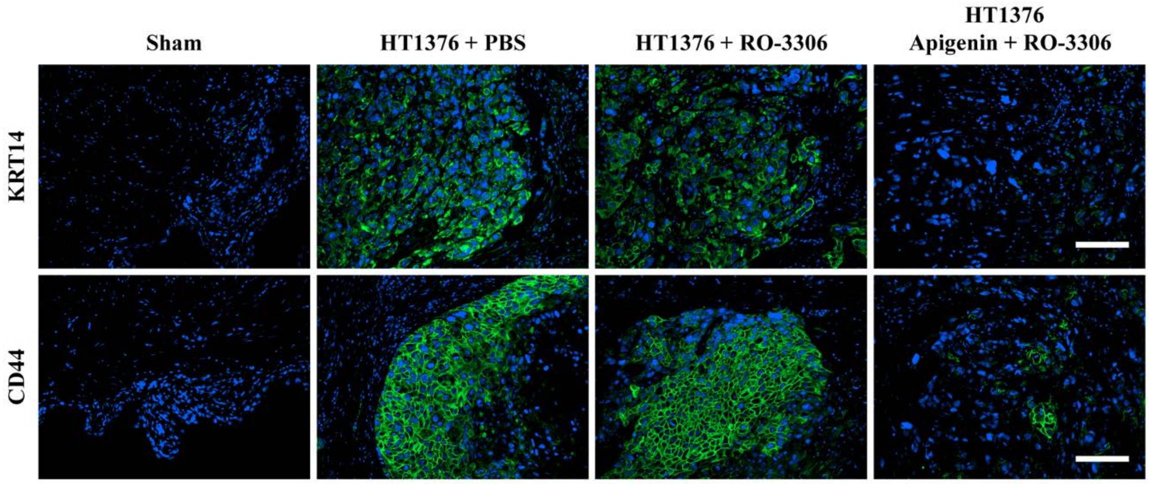

Supplementary Fig. 11 Immunostaining of BC stem cell markers in xenograft tumor samples.

Immunofluorescence of BC stem cell markers (green) including KRT14 (upper panel) and CD44 (lower panel) in xenograft tumors treated with RO-3306 (4 mg/kg) alone or combined with apigenin (50 mg/kg). PBS was used as the vehicle control. Representative images are shown at ×200 magnification. Scale bars = 100 µm. Nuclei were stained with DAPI (blue).

SUPPLEMENTARY TABLES

Supplementary Table 1. Information of ORF constructs

ORF constructs	Source	Identifier
human *TFCP2L1* in pBluescript	Dharmacon	MHS6278-202806269
human *ID2* in pDONR223	Addgene	82960
human pLKO.1 *ID2*	Sigma-Aldrich	TRCN0000021067

Supplementary Table 2. Oligonucleotides for shRNA

Oligonucleotides (shRNA)	Target sequence
human *TFCP2L1*	GCTCTTCAACGCCATCAAAGG
human *ID2*	CCCACTATTGTCAGCCTGCAT

Supplementary Table 3. Primer information used in the RQ-PCR analysis

Oligonucleotides (qPCR)	Forward primer	Reverse primer
human *TFCP2L1*	GCTCTTCAACGCCATCAA	CAGGGGCACTCGATTCTG
human *ID2*	CAGCATCCCCCAGAACAAGAA	CGATCTGCAGGTCCAAGATGT

Supplementary Table 4. Primer information used in the ChIP-qPCR analysis

Oligonucleotides (qChIP)	Forward primer	Reverse primer
human *ID2*_#1	CTCCGATGGGTTGCAGTGAA	CGGCAGCTCTAAAATCACAGCTA
human *ID2*_#2	TGCAGCACGTCATCGACTACAT	CTGGTGATGCAGGCTGACAA

SUPPLEMENTARY REFERENCES

1. Robertson, A. G. *et al.* Comprehensive Molecular Characterization of Muscle-Invasive Bladder Cancer. *Cell* **171**, 540-556 e525 (2017).
2. Heo, J. *et al.* Phosphorylation of TFCP2L1 by CDK1 is required for stem cell pluripotency and bladder carcinogenesis. *EMBO Mol. Med.* **12**, e10880 (2020).

UNCROPPED WESTERN BLOT RESULTS

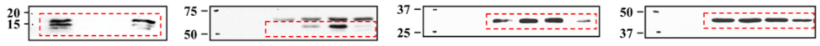

Fig. 2b (ID2 WB) | Fig. 2b (TFCP2L1 WB) | Fig.2b (CDK1 WB) | Fig. 2b (β-ACTIN WB)

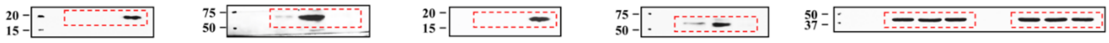

Fig. 2e (HT1197_ID2 WB) | Fig. 2e (HT1197_TFCP2L1 WB) | Fig. 2e (HT1376_ID2 WB) | Fig. 2e (HT1376_TFCP2L1 WB) | Fig. 2e (HT1197(Left)_HT1376(Right)_ β-ACTIN WB)

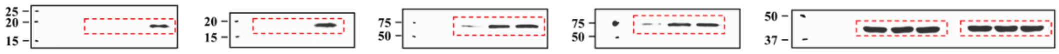

Fig. 3a (HT11 97_ID2 WB) | Fig. 3a (HT1376_ID2 WB) | Fig. 3a (HT11 97_TFCP2L1 WB) | Fig. 3a (HT1376_TFCP2L1 WB) | Fig. 3a (HT1197(Left), HT1376(Right)_ β-ACTIN WB)

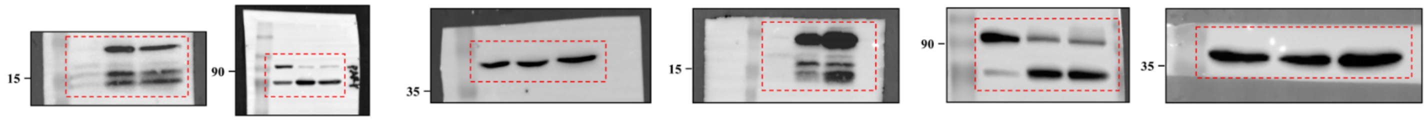

Fig. 4b (HT1376_Cleaved Caspase 3 WB) | Fig. 4b (HT1376_PARP WB) | Fig. 4b (HT1376_ β-ACTIN WB) | Fig. 4b (HT1197_Cleaved Caspase 3 WB) | Fig. 4b (HT1197_PARP WB) | Fig. 4b (HT1376_ β-ACTIN WB)

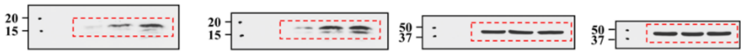

Fig. 5f (HT1197_ID2 WB) | Fig. 5f (HT1376_ID2 WB) | Fig. 5f (HT1197_β-ACTIN WB) | Fig. 5f (HT1376_β-ACTIN WB)

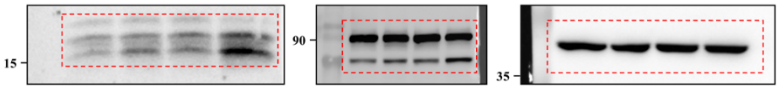

Fig. 6b (HT1376_Cleaved Caspase 3 WB) | Fig. 6b (HT1376_PARP WB) | Fig. 6b (HT1376_ β-ACTIN WB)

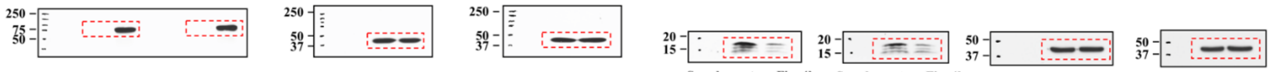

Supplementary Fig. 3a (HT1197(Left), HT1376(Right)_Flag WB) | Supplementary Fig. 3a (HT1197(L)_ β-ACTIN WB) | Supplementary Fig. 3a (HT1376(R)_ β-ACTIN WB) | Supplementary Fig. 4b (5637_ID2 WB) | Supplementary Fig. 4b (RT4_ID2 WB) | Supplementary Fig. 4b (5637_ β-ACTIN WB) | Supplementary Fig. 4b (RT4_ β-ACTIN WB)

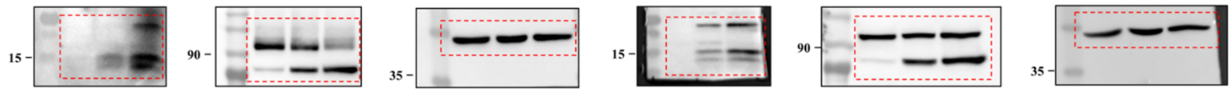

Supplementary Fig. 5b (HT1376_Cleaved Caspase 3 WB) | Supplementary Fig. 5b (HT1376_PARP WB) | Supplementary Fig. 5b (HT1376_ β-ACTIN WB) | Supplementary Fig. 5b (HT1197_Cleaved Caspase 3 WB) | Supplementary Fig. 5b (HT1197_PARP WB) | Supplementary Fig. 5b (HT1197_ β-ACTIN WB)

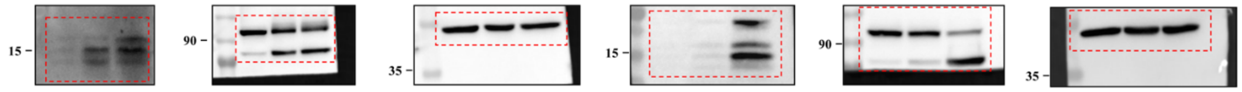

Supplementary Fig. 6b (HT1376_Cleaved Caspase 3 WB) | Supplementary Fig. 6b (HT1376_PARP WB) | Supplementary Fig. 6b (HT1376_ β-ACTIN WB) | Supplementary Fig. 6b (HT1197_Cleaved Caspase 3 WB) | Supplementary Fig. 6b (HT1197_PARP WB) | Supplementary Fig. 6b (HT1197_ β-ACTIN WB)

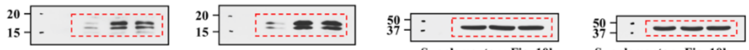

Supplementary Fig. 10b (HT1197_ID2 WB) | Supplementary Fig. 10b (HT1376_ID2 WB) | Supplementary Fig. 10b (HT1197_β-ACTIN WB) | Supplementary Fig. 10b (HT1376_β-ACTIN WB)
